# MicroRNAs in periodontal disease: from pathogenic mechanisms and RANKL/OPG regulation to nanoparticle delivery and personalized medicine

**DOI:** 10.3389/fimmu.2026.1876885

**Published:** 2026-07-10

**Authors:** Peiru Yang, Zhulin Li, Sisi Li, Bochao Wu, Anwen Lei, Dandan Ren, Jue Zhang, Chao Deng, Mingzhao Wang, Songlin Zhou

**Affiliations:** 1 Anhui Engineering Research Center for Oral Materials and Application, Wannan Medical University, Wuhu, China; 2 College of Oral Medicine, Wannan Medical University, Wuhu, China; 3 Xuancheng City People’s Hospital, Xuancheng, Anhui, China; 4 Hefei Stomatological Hospital, Hefei, Anhui, China

**Keywords:** biomarkers, microRNA, nanoparticle delivery, PDLSC differentiation, periodontitis, personalized medicine, RANKL/OPG axis

## Abstract

Dysregulated host-microbe interactions are a hallmark of periodontal disease (PD), a chronic inflammatory condition that causes progressive alveolar bone resorption and tooth loss. MicroRNAs (miRNAs) have become important epigenetic regulators, offering new ways to understand disease pathophysiology and to provide tailored treatments. The current analysis uniquely synthesizes pathogen-specific miRNA signatures, modulation of the Receptor Activator of Nuclear Factor Kappa-B Ligand (RANKL)/Osteoprotegerin (OPG) axis, periodontal ligament stem cells (PDLSCs) osteogenesis, and enhanced delivery platforms into a coherent precision medicine framework, in contrast to other studies that examined these areas independently. We investigate the role of miRNAs in PD, assess their potential as non-invasive diagnostic biomarkers, and review novel therapeutic approaches targeting these molecules. *Porphyromonas gingivalis* and other periodontal infections cause aberrant miRNA expression that accelerates bone loss by impairing the development of PDLSCs, disrupting the RANKL/OPG axis, and sustaining pro-inflammatory cytokine cascades (IL-1, IL-6, TNF-α). Based on replication across many independent investigations, three of the many identified miRNAs—miR-146a, miR-155, and miR-223—show the most promise for diagnosis and treatment. These miRNAs, found in saliva and gingival crevicular fluid, serve as reliable non-invasive indicators. Preclinical studies show that anti-miR inhibitors and miRNA mimics, administered via hydrogels or nanoparticles, successfully reduce inflammation and promote alveolar bone repair. However, several issues remain unresolved, including miRNA instability, off-target effects, and interpatient variability, as well as contradictory results from multiple studies, such as the opposing functions of miR-21 across various cell types. In summary, targeted host modification by miRNA-based therapies is a paradigm change from traditional symptomatic therapy. To incorporate these strategies into clinical practice and eventually enable regenerative and customized periodontal treatment, it will be crucial to address current delivery and safety constraints through improved nanocarriers and patient-specific profiling.

## 1 Introduction

Periodontal disease (PD) is a chronic inflammatory condition affecting the gingiva, periodontal ligament (PDL), cementum, and alveolar bone. Dysbiotic microbial communities primarily initiate it within the subgingival biofilm. The “red complex” pathogens, *Porphyromonas gingivalis* (*P. gingivalis*), *Tannerella forsythia* (*T. forsythia*), and *Treponema denticola*, possess potent virulence factors, including lipopolysaccharides (LPS) (triggering robust inflammatory responses), gingipains (degrading host tissues and evading immune defenses), and fimbriae/adhesins (promoting biofilm formation). However, PD pathogenesis is not solely attributable to microbial aggression; rather, it is predominantly driven by the host’s immune-inflammatory response (1, 2). The process progresses from microbial colonization and biofilm formation to gingivitis, then to periodontitis, in which chronic inflammation irreversibly destroys the PDL and alveolar bone (3). Without treatment, the disease advances to periodontal pockets, alveolar bone resorption, tooth mobility, and eventual tooth loss, severely affecting 10–12% of the population (4).

The extent of immune engagement and clinical presentation of periodontitis varies among individuals and may be influenced by environmental, genetic, and comorbid factors (5). Given that periodontitis affects approximately 11.2% of adults worldwide with significant systemic health consequences, researchers are actively seeking novel signaling molecules for both diagnosis and treatment (6).

Although several thorough reviews have recently examined miRNAs in PD, including Saha 2026 and Fayazi 2025 (7, 8), none have methodically integrated advanced delivery platforms, Receptor Activator of Nuclear Factor Kappa-B Ligand (RANKL)/Osteoprotegerin (OPG) axis regulation, PDL Stem Cell (PDLSC) osteogenesis, and pathogen-specific miRNA signatures into a coherent precision medicine framework. Prioritizing miRNAs with the greatest diagnostic and therapeutic potential based on replication across multiple studies and functional validation, the current study uniquely synthesizes these domains to propose actionable routes for clinical translation. We provide a critical comparison of contradictory results, such as miR-21 showing contrasting effects in Bone Marrow Mesenchymal Stem Cells (BMSCs) vs PDLSCs, a prioritized ranking of important miRNA candidates, and specific connectivity between mechanistic targets and their related delivery mechanisms, in contrast to previous broad-scope assessments.

Epigenetic mechanisms, particularly microRNA (miRNA) molecules, play a central role in regulating inflammation and host defense. miRNAs are small, single-stranded non-coding RNA molecules that control more than 60% of human genes (9). They regulate post-transcriptional gene expression by binding to the 3′UTR of target mRNAs, blocking translation or degrading the mRNA. miRNAs are transcribed from specialized units and processed by endonucleases, including Drosha/DiGeorge Syndrome Critical Region 8 (DGCR8) and Dicer, ultimately incorporating into the RNA-induced silencing complex (RISC). miRNA-gene networks regulate stem cell self-renewal, proliferation, migration, apoptosis, immune modulation, and differentiation (10). Since their discovery in 1993, miRNAs have been extensively studied in bone metabolism, particularly in RANKL-induced osteoclastogenesis, a central process in bone loss. Inflammatory mediators upregulate RANKL expression in periodontal resident cells (gingival fibroblasts and PDL cells (PDLCs)), suggesting that specific miRNAs govern this pathogenic axis (11).

miRNAs regulate innate and adaptive immune responses involving T cells, B cells, neutrophils, macrophages, and dendritic cells. miR-146a, miR-29, miR-15, miR-148, and miR-223 are upregulated in periodontitis, while miR-31, miR-92, and miR-451 are downregulated (12). Among these, miR-223 has repeatedly demonstrated diagnostic promise, with miR-142-3p and miR-146a also indicating disease activity. The stability of miRNAs in gingival crevicular fluid (GCF) and their association with disease traits support their utility as non-invasive biomarkers for early detection (13). However, further investigation is required to clarify the roles of miRNA in the homeostasis and pathophysiology of periodontal tissue (14).

PDLSCs are essential for maintaining periodontal health because they differentiate into fibroblasts, osteoblasts, and cementoblasts, all of which are derived from neural crest-derived progenitors. PDLSCs undergo osteogenic differentiation, produce chemokines, and decrease inflammation, as first reported by Seo et al. (15). Nevertheless, the regenerative capacity of PDL-derived cells is diminished by the inflammatory periodontal microenvironment (16). Therefore, altering PDL-derived cell biological activity under inflammatory stress is a major therapeutic objective (17). The poor clinical success of traditional therapies like guided bone regeneration and guided tissue regeneration (GTR) highlights the need for new techniques, including miRNA-based delivery methods (18).

Precision medicine in periodontology aims to tailor treatment, prevention, and detection by integrating biological, genetic, epigenetic, behavioral, and environmental factors (19). This approach addresses limitations of standard practices that do not consistently account for variable disease progression across patients. Diagnostic tools combining biomarkers (IL-1β, salivary and GCF proteomics, and miRNAs) with digital platforms have demonstrated improved accuracy and earlier disease detection (20). Personalized interventions, including host-modulating medications and customized antibiotics, have enhanced clinical outcomes, while preventive programs informed by genetic, systemic, and behavioral risk assessments have reduced tooth loss (21). By integrating omics technologies, real-time diagnostics, and behavioral insights, precision periodontology advances patient-centered care, enabling more accurate diagnoses, superior treatment outcomes, and sustained protection (22).

With an emphasis on host immune regulation, osteoclastogenesis via the RANKL/OPG axis, and PDLSC osteogenic differentiation, this review critically analyzes current studies on the regulatory involvement of miRNAs in PD pathogenesis. In the context of the developing field of precision periodontal medicine, we also assess the potential of miRNAs as non-invasive diagnostic biomarkers in saliva and GCF and investigate novel miRNA-based therapeutic approaches, including miRNA mimics, anti-miR inhibitors, and sophisticated nanoparticle (NP)- or hydrogel-based delivery systems. Throughout, we address conflicting data, prioritize miRNAs with reproducible results, and clearly connect mechanistic discoveries to therapeutic possibilities.

## 2 MicroRNA biogenesis and functions

Understanding miRNA biogenesis and function is essential for elucidating their roles in cell biology and human disease (23). miRNA production begins in the nucleus, where RNA polymerase II transcribes specific genes, generating long primary miRNAs (pri-miRNAs) that fold into characteristic hairpin-loop structures (24). The nuclear microprocessor complex, composed of the RNase III nuclease Drosha and the dsRNA-binding protein DGCR8, recognizes and cleaves these pri-miRNAs, releasing the precursor miRNA (pre-miRNA), a about 70-nucleotide hairpin (25). Following export to the cytoplasm via Exportin-5 and RanGTP, the pre-miRNA is cleaved by Dicer into a mature double-stranded RNA of approximately 22 base pairs. This second RNase III enzyme associates with the 3′ overhang and double-stranded stem (26, 27).

Dicer serves as a “molecular ruler” throughout this procedure. After that, one of the two strands is added to the RISC, which is made up of an Argonaute protein—in humans, Argonaute 2 (AGO2). The mature miRNA directs RISC to complementary sequences, which are usually found in the 3′-UTR of target mRNAs, using its “seed region” (nucleotides 2–7) (28). RISC mediates post-transcriptional gene silencing by either blocking translation and destabilizing the mRNA (when pairing is incomplete) or cleaving the mRNA (when pairing is complete or near-complete) (29). These structural insights, including how the distance of the PAZ domain from Dicer’s catalytic site affects cleavage accuracy and how AGO2 conformational changes enable “walking” along target mRNAs, have facilitated diagnostic and therapeutic applications of miRNAs (30).

In addition to their typical cytoplasmic functions, miRNAs and AGO proteins can influence transcription, splicing, and chromatin modification in the nucleus and mitochondria. Target-directed miRNA degradation by ZSWIM8, post-translational alterations (phosphorylation, ubiquitination), and epitranscriptomic modifications (A-to-I editing, m6A, 3′ uridylation) that adjust synthesis, stability, and target selectivity further control miRNA activity (31).

**Figure 1:**
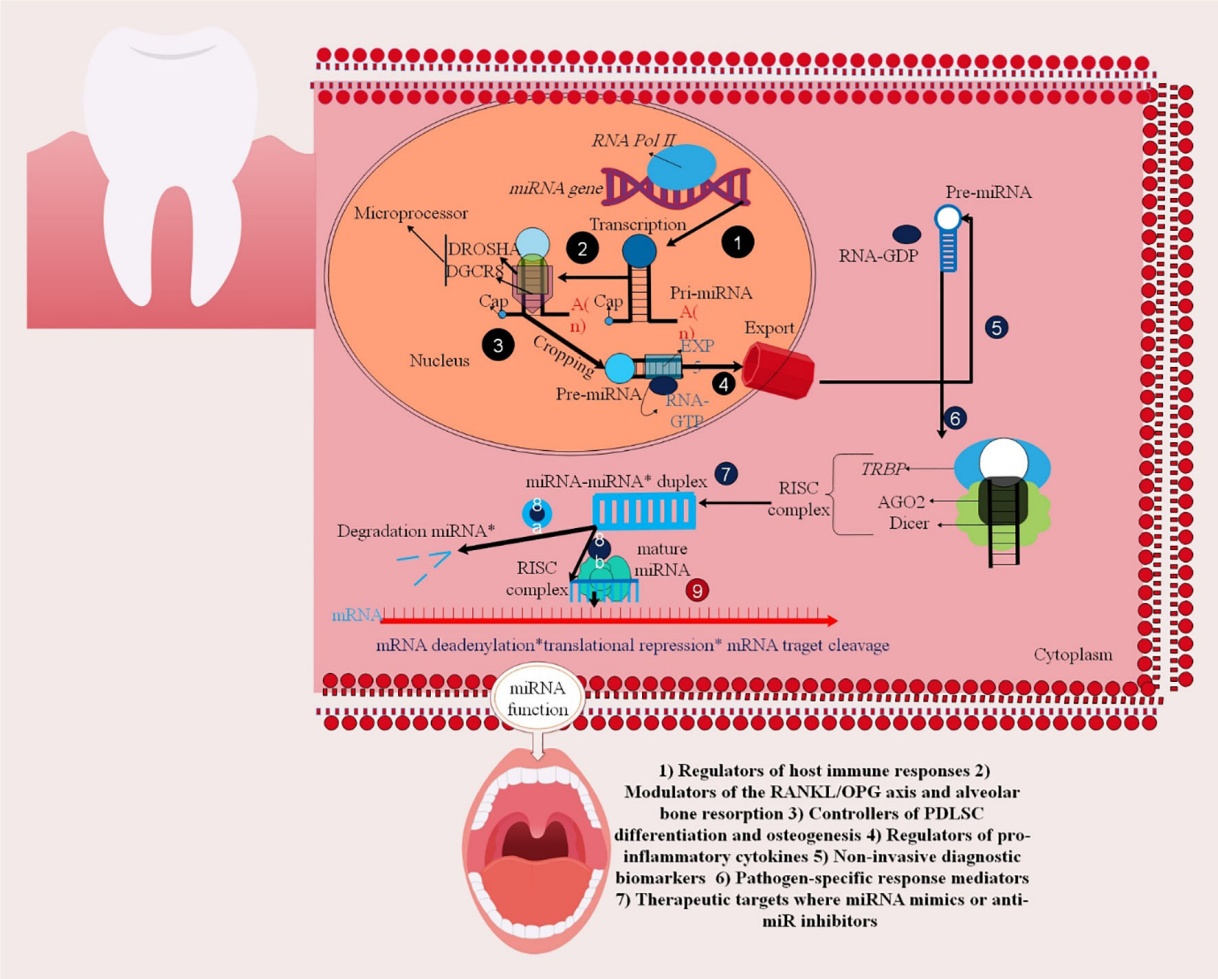
Schematic depiction of miRNA production and their diverse roles in PD. The left panel depicts the classic miRNA biogenesis pathway: miRNA genes are transcribed by RNA polymerase II (Pol II) into pri-miRNAs, which form a hairpin structure with a 5′ cap and a 3′ poly **(A)** tail. Within the nucleus, the Microprocessor complex (DROSHA and DGCR8) cleaves the pri-miRNA to produce the approximately 70-nucleotide pre-miRNA. After exportin-5/Ran-GTP-facilitated transport to the cytoplasm, Dicer, in association with Trans-Activation Response RNA-Binding Protein, converts the pre-miRNA into a mature ~22-base-pair miRNA-miRNA* duplex. The guide strand is specifically incorporated into the RISC that includes AGO2, whereas the passenger strand (miRNA) is subjected to degradation. The mature RISC complex subsequently associates with complementary regions in the 3 ′ UTRs of target mRNAs, leading to mRNA deadenylation, translational repression, or mRNA cleavage. The right panel delineates the seven principal functions of miRNAs in periodontitis: (1) regulators of host immune responses (macrophages, neutrophils, T/B cells); (2) modulators of the RANKL/OPG axis and alveolar bone resorption; (3) controllers of PDLSC differentiation and osteogenesis; (4) regulators of pro-inflammatory cytokines (IL-1, IL-6, TNF-α); (5) non-invasive diagnostic biomarkers in saliva and GCF; (6) pathogen-specific response mediators induced by periodontal bacteria; and (7) therapeutic targets for miRNA mimics and anti-miR inhibitors in periodontal treatment.

Although the discovery of microRNAs and their gene-regulatory mechanisms was recognized with the 2024 Nobel Prize awarded to Victor Ambros and Gary Ruvkun, miRNA-based treatments have not yet achieved the same level of clinical success as siRNAs due to issues such as potential immunogenicity, poor delivery efficiency, and modest effects on multiple targets. However, miRNAs show great promise as diagnostic indicators (32) (**Figure 1**).

## 3 The role of the miRNAs in periodontitis

The severity and progression of PD depend on the dynamic equilibrium between microbial dysbiosis and the host immune response, which comprises innate and adaptive immunity (33). By modulating chemokine mRNA stability, miRNAs regulate neutrophil function, including adhesion to and migration from blood vessels into inflamed tissues (34). Bacterial plaque components can dysregulate miRNAs, potentially rendering the innate and adaptive immune systems either hyporesponsive to microbial changes or hyperresponsive, leading to excessive tissue degradation (35).

Among the most consistently replicated findings, six members of the miR-200 family (miR-200a, -200b, and -200c, both -3p and -5p strands) were significantly elevated in the gingival epithelium of chronic periodontitis (CP) patients compared to healthy controls, whereas miR-141 and miR-429 showed no differential expression. The combined miR-200 panel demonstrated high diagnostic accuracy (Area Under the Curve (AUC) = 0.997, 99% sensitivity and specificity) and a positive correlation with disease severity, supporting its potential as a non-invasive biomarker for the detection of early periodontitis (36). Nonetheless, this work lacked an independent validation cohort, and the very high AUC may reflect overfitting. Before clinical use, further replication in larger, multi-center populations is necessary.

### 3.1 Expression profiles of miRNAs in periodontal tissues

Numerous illnesses have been linked to dysregulated miRNA expression, making miRNAs attractive candidates for biomarkers. MiRNAs affect immunological responses and the function of periodontal cells, such as PDLCs and oral epithelial cells, in periodontitis (8, 37). Future studies should focus on refining cell-type-specific expression patterns, clarifying regulatory mechanisms, and building bioinformatic networks to guide the development of diagnostic and therapeutic approaches, as miRNA expression profiles vary between healthy and diseased gingival tissues (38). **Table 1** summarizes sample sizes, key results, diagnostic performance (where reported), and methodological limitations in a review of research on miRNA expression in periodontal tissues.

**Table 1:** Summary of miRNA expression studies in periodontal tissues.

Study (Ref)	Sample type	Sample size	Key miRNA findings	Diagnostic performance (AUC)	Limitations
Buragaite-Staponkiene 2023 (39)	Gingival tissue, saliva, plasma	Not specified	Increased miR-140-3p, increased miR-145-5p, decreased miR-125a-3p in PD	Not reported	No validation cohort; confounders (RA, biologics)
Amaral 2019 (40)	Gingival tissue	CP *vs.* Aggressive Periodontitis (AP)	No significant differences between CP and AP; most abundant: miR-1274b, let-7b-5p, miR-24-3p	Not applicable	Small sample; no healthy control comparison
Guzeldemir-Akcakanat 2023 (41)	Oral tissue	25 severe PD *vs.* 25 healthy	Increased miR-223-3p, miR-203b-5p, miR-146a-5p, miR-155-5p; decreased miR-185-5p, miR-21-3p, miR-17-3p	Not reported	Moderate sample size; no validation
Öngöz Dede 2023 (42)	Saliva	90 (30 PD, 30 gingivitis, 30 healthy)	Increased miR-203, miR-142-3p, miR-146a, miR-146b, miR-155 in PD; increased smoking miR-142-3p	Not reported	Short follow-up; non-surgical periodontal therapy (NSPT) effect non-significant
Almiñana-Pastor 2023 (43)	GCF	12 healthy *vs.* 11 severe CP	miR-199, miR-146a, miR-30a, miR-338; miR-199 most accurate	Not specified for individual miRNAs	Very small sample (n=11-12); no validation
Chen 2025 (44)	Exosomes (EXOs) (Dental Follicle Stem Cells (DFSCs)/PDLSCs)	Mouse model + *in vitro*	Decreased miR-184 in LPS-Derived EXOs (L-D-EXOs); antagomir reduced oxidative stress	Not applicable	Animal model; human validation needed
Baru 2025 (45)	Not specified	50 (17 PD, 33 healthy)	Increased miR-29b-3p, miR-34a-5p, miR-155-5p, miR-181a-5p, miR-192-5p	0.69-0.74 (miR-192 best)	Moderate AUC; no validation cohort
Guo 2026 (46)	GCF	80 CP *vs.* 100 healthy	Increased miR-486-5p; targets Phosphatase and Tensin Homolog (PTEN); suppresses PDLC proliferation, promotes apoptosis	0.878	Cross-sectional; functional validation only *in vitro*
Venugopal 2025 (47)	Not specified	Case-control	Six dysregulated miRNAs (miR-146a-5p, miR-125a-5p, miR-20a-5p, miR-155-5p, miR-196a-5p, miR-499a-5p); co-expression pattern identified	Not reported	Validation needed for co-expression panel

A number of important findings emerge from the evaluated research (Guo et al.;^39^–^42^, ^44^, ^45^, ^47^). First, the most consistently elevated miRNAs across multiple cohorts are miR-146a, miR-155, and miR-223, supporting their selection as potential biomarkers. Second, diagnostic performance varies significantly, with AUC values ranging from 0.69 to 0.878. Because of potential overfitting, the highest reported AUC (0.997 for the miR-200 family; Section 3) requires independent confirmation. Third, significant methodological variation makes direct comparisons across research difficult (Guo et al.;^39^–^42^, ^44^, ^45^, ^47^).

Several studies need particular discussion on their strengths and weaknesses. Periodontitis-associated miRNAs were discovered in gingival tissue, saliva, and plasma by Buragaite-Staponkiene et al. (39). They noted that biologic disease-modifying antirheumatic drugs altered gingival miRNA profiles, a significant confounding factor often disregarded (39). Despite differences in clinical manifestations, Amaral et al. (40) found no significant differences in miRNA expression patterns between aggressive and CP (p > 0.05), indicating shared pathogenic pathways. However, determining whether both disease types share dysregulation in relation to health is limited by the lack of healthy controls (40).

In severe periodontitis, Guzeldemir-Akcakanat et al. (41) found that inflammatory miRNAs (miR-223-3p, miR-146a-5p, and miR-155-5p) were upregulated while miR-185-5p, miR-21-3p, and miR-17-3p were downregulated. Notably, although functional validation was not performed, the authors suggested MZB1 as a target of miR-185. Examining salivary miRNA levels in smokers and non-smokers, Öngöz Dede et al. (42) discovered that smoking markedly increased miR-142-3p levels in all groups—a crucial illustration of environmental control of miRNA expression. Short-term intervention may not correct miRNA dysregulation, as evidenced by the lack of a statistically significant improvement following NSPT (42).

High-throughput miRNA sequencing of GCF from only 11 CP patients and 12 healthy controls was used by Almiñana-Pastor et al. (43), a relatively small sample size that limits generalizability. Despite this, they identified miR-199 as a potential biomarker; however, AUC values for specific miRNAs were not reported (43). Chen et al. (44) demonstrated that miR-184 antagomir decreased oxidative stress and enhanced bone regeneration in a mouse periodontitis model via the Peroxisome Proliferator-Activated Receptor Alpha (PPARα)-Akt-JNK pathway, providing functional confirmation of miR-184 in Dental Follicle Stem Cell (DFSC)-derived EXOs. Although human translation is still forthcoming, this serves as an example of thorough mechanistic validation (44).

A five-miRNA panel with AUCs ranging from 0.69 to 0.74 was reported by Baru et al. (45), suggesting limited diagnostic accuracy. Clinical application is limited by the small sample size (50 overall, only 17 with periodontitis) and the absence of an independent validation cohort. On the other hand, this research examined the expression of miR-486-5p in 100 healthy controls and 80 patients with CP. GCF from CP patients showed a substantial increase in miR-486-5p, which also showed diagnostic significance (AUC = 0.878). MiR-486-5p overexpression was shown to inhibit cell proliferation, induce apoptosis, and activate the release of inflammatory cytokines in LPS-stimulated PDLCs *in vitro*. Mechanistically, miR-486-5p was shown to directly target PTEN. The authors conclude that miR-486-5p is a useful biomarker for CP diagnosis and that its overexpression is associated with disease progression (Guo et al.).

A five-miRNA co-expression pattern (miR-146a-5p, miR-125a-5p, miR-155-5p, miR-20a-5p, and miR-196a-5p) that separates generalized periodontitis from health was discovered by Venugopal et al. (47). This profile implicates the IL-1 pathway and epithelial-mesenchymal transition. Although theoretically encouraging, the co-expression pattern must be validated in a separate cohort, and the study did not provide diagnostic accuracy measures (47).

A critical evaluation of the existing data indicates several significant shortcomings present in almost every study examining miRNA expression in periodontal tissues. These include: (1) very small sample sizes (typically 11–33 patients per group); (2) lack of independent validation cohorts; (3) cross-sectional designs that prevent causal inference; (4) significant methodological heterogeneity (sample type, normalization, miRNA panels); (5) emphasis on inflammatory miRNAs (miR-146a, miR-155) with relative neglect of miRNAs related to osteogenesis or oxidative stress; (6) limited functional validation (only miR-184 and miR-486-5p have been mechanistically validated); and (7) insufficient control for confounders like smoking, chronic diseases, and medications).

The most promising diagnostic miRNA candidates, with consistent evidence across many studies, according to this rigorous review, are miR-146a, miR-155, and miR-223. Although independent confirmation is needed, the miR-200 family (Section 3) and miR-486-5p exhibit good diagnostic accuracy. Large longitudinal cohorts with standardized procedures (such as miRNeasy-based separation, U6 or cel-miR-39 normalization), multi-omics integration, functional validation in pertinent cell types, and investigation of exosomal miRNAs as non-invasive biomarkers should be given top priority in future research.

### 3.2 Dysregulation of miRNAs in response to periodontal pathogens (*P. gingivalis*)

MicroRNAs regulate multiple processes associated with inflammatory diseases and infections. Bacterial infection alters miRNA expression, potentially impairing host defense mechanisms (48).

*P. gingivalis* is considered a primary pathogen in periodontitis, a disease driven by dysbiosis of oral polymicrobial communities. The host defense against infectious agents initiates inflammatory signaling cascades, in which miRNAs function as post-transcriptional regulators (49). miRNA expression profiles differ between diseased and healthy tissues, suggesting their utility as diagnostic or prognostic tools. Specific miRNA species (miR-128, miR-146, miR-203, and miR-584) play regulatory roles in innate immunity, indicating potential therapeutic targets. *P. gingivalis* and its LPS may modulate host miRNA function and downstream mRNA targets (50).

*Streptococcus gordonii* (*S. gordonii*), a commensal bacterium that promotes colonization by other diseases, was introduced into C57BL/6J mice by Aravindraja et al. (37). Alveolar bone loss in the mandible and maxilla was seen in infected mice. Only 10 miRNAs were elevated and 32 downregulated at 16 weeks, indicating early widespread dysregulation followed by stability. At 8 weeks, 191 miRNAs were increased, and 22 were downregulated. While miR-2135 and miR-145 were consistently elevated, miR-210 and miR-423-5p were consistently downregulated. MiR-22, miR-30c, miR-720, and miR-339-5p were identified as infection predictors using machine learning (37). The single-species model and the lack of external validation of machine learning results are among its drawbacks.

To corroborate alveolar bone resorption and IgG responses, Aravindraja et al. (37) studied miRNA expression in *P. gingivalis*-infected animals at 8 and 16 weeks. Twenty-six miRNAs were upregulated and fourteen downregulated at eight weeks; seven were upregulated, and just one was downregulated at sixteen weeks. There was constant upregulation of miR-103 and miR-30d. A significant difference between LPS-separated and live bacterial infection was highlighted by the observation that miRNAs identified in *in vitro* LPS experiments were absent *in vivo*. Phagocytosis, bacterial invasion, and endocytosis were identified via pathway analysis. In human periodontitis tissues, four elevated miRNAs (miR-31, miR-125b, miR-15a, and miR-195) were also found, providing cross-species confirmation. Periodontitis and systemic illness may be connected by miR-103 and miR-30d, which are likewise increased in diabetes and hypercholesterolemia (51).

Using gingival tissue from 64 individuals (healthy, periodontitis alone, Type 2 Diabetes Mellitus (T2DM) alone, and periodontitis+T2DM), Mathews et al. (52) evaluated the role of miR-155 on macrophage polarization. While Arg-1 (M2 marker) was greatest in healthy controls, the periodontitis+T2DM group had the maximum miR-155-fold change (11.17) and TNF-α expression (10.11), suggesting M1 macrophage polarization. While miR-155 performed poorly, TNF-α had limited diagnostic effectiveness (AUC = 0.74) (52). Crucially, there is insufficient functional validation in human samples, and the cross-sectional design prevents causal inference.

The roles of miR-26a-5p and miR-26b-5p in the pathophysiology of PD were investigated by Uttamani et al. (53). Both miRNAs in sick gingival tissues recovered to normal levels four to six weeks after non-surgical therapy, demonstrating reversibility. Phospholipase C Beta 1 (PLCB1) was identified as a new target. Upon pathogen activation, transfection with miR-26a-5p decreased cell migration and increased cytokine production. The scientists concluded that downregulation of miR-26a-5p would hinder wound healing and host defense (53). Post-treatment reversibility is one of its strengths; *in vitro*-only functional validation and a small sample size are its drawbacks.

Aravindraja et al. (54) observed mandibular bone loss in mice infected with *T. forsythia*. 115 differentially expressed miRNAs (99 downregulated) were found by NanoString analysis. 67 miRNAs (miR-375, miR-200c, miR-200b, and miR-141) were downregulated at 8 weeks. At 16 weeks, 32 miRNAs were downregulated (miR-2135, miR-720) while 16 miRNAs were upregulated (miR-let-7c, miR-146a). Human periodontitis tissues also showed dysregulated levels of miRNAs (miR-200b, miR-141, miR-205, and miR-146a). Although these signals lack external validation, machine learning identified miR-592-5p and miR-339-5p as predictors of infection (54). **Table 2** illustrates that various periodontal infections elicit unique miRNA expression profiles.

**Table 2:** Alterations in pathogen-specific miRNA expression and their functional implications in periodontitis.

Pathogen	Model system	Time points	Key miRNA findings	Machine learning identified	Limitations	REF.
*S. gordonii*	C57BL/6J mice	8 and 16 weeks	At 8 weeks: 191 miRNAs upregulated, 22 downregulated; at 16 weeks: 10 upregulated, 32 downregulated; miR-210 and miR-423-5p consistently downregulated	miR-22, miR-30c (8wk); miR-720, miR-339-5p (16wk)	Single-species model; does not reflect human polymicrobial biofilm	(37)
*P. gingivalis*	C57BL/6J mice	8 and 16 weeks	At 8 weeks: 26 upregulated, 14 downregulated; at 16 weeks: 7 upregulated, 1 downregulated; miR-103 and miR-30d upregulated at both time points	Not reported	*In vitro* LPS-induced miRNAs not detected *in vivo*; human validation limited	(51)
*P. gingivalis* + T2DM	Human gingival tissue (64 patients)	Cross-sectional	miR-155 upregulated 11.17-fold in periodontitis+T2DM; M1 polarization	AUC = 0.74 (TNF-α)	Cross-sectional; modest diagnostic efficacy for miR-155	(52)
*T. forsythia*	C57BL/6J mice	8 and 16 weeks	At 8 weeks: 67 downregulated (miR-375, miR-200c, miR-200b); at 16 weeks: 16 upregulated (miR-let-7c, miR-146a), 32 downregulated	miR-592-5p, miR-339-5p	Single-species model; machine learning findings lack external validation	(54)

Every study on miRNAs produced by pathogens has several caveats. The first is that human polymicrobial periodontitis cannot be simulated in mouse models using a single species. Second, there is no third-party verification of machine-learning-generated signatures (*e.g.*, miR-592-5p, miR-339-5p). Thirdly, functional validation is often absent from research. The fourth point is that there is a contradiction between the results obtained using pure LPS and those obtained using live bacterial infection. Aravindraja et al. (51) found no miRNAs generated by LPS *in vitro*, whereas Uttamani et al. (53) were able to employ LPS effectively *in vitro*. Fifteenth, it is uncommon to control confounding variables like smoking, systemic illness, or drugs.

The following miRNAs have been validated across various models or species and are pathogen-responsive: miR-146a (*P. gingivalis*, *T. forsythia*, human tissues), miR-155 (*P. gingivalis*+T2DM, human periodontitis), the miR-200 family (*T. forsythia*, human tissues), and miR-210 (*S. gordonii*, *T. forsythia*). Prioritizing polymicrobial models, validating ML signatures with separate cohorts, and functional validation using CRISPR or antagomirs should be the focus of future investigations.

### 3.3 miRNA-mediated regulation of pro-inflammatory cytokines (IL-1, IL-6, TNF-α)

MiRNAs regulate inflammation by modulating their expression in immune cells, thereby influencing the initiation, progression, and resolution of inflammation through positive and negative feedback loops (55). One of the most extensively characterized pro-inflammatory miRNAs is miR-155, which is activated by LPS via the Myeloid Differentiation Primary Response 88 (MyD88) and TIR-Domain-Containing Adapter-Inducing Interferon-β pathways and promotes TNF-α translation. miR-155-deficient mice exhibit attenuated immune responses, while overexpression enhances B-cell germinal center formation (56). But it’s crucial to remember that miR-155 also has anti-inflammatory effects in certain situations, including when it targets SH2-Containing Inositol Phosphatase 1 (SHIP1) and Suppressor of Cytokine Signaling (SOCS)-1 in macrophages, highlighting its context-dependent function.

Several additional miRNAs promote inflammation through diverse mechanisms. miR-92a, upregulated in atherogenic endothelial cells, is transferred to macrophages via extracellular vesicles (EVs), where it upregulates pro-inflammatory genes (56). By suppressing Zeb-1 and increasing cyclooxygenase-2 and Monocyte Chemoattractant Protein-1 (MCP-1) expression, the miR-200 family promotes inflammation. While miR-27a displays a similar phenotype by targeting Interferon Regulatory Factor 4 and PPAR-γ, miR-29c causes pro-inflammatory effects in diabetic nephropathy by targeting tristetraprolin. miR-23a activates NF-κB by targeting A20 and inhibits the anti-inflammatory JAK1/STAT6 pathway by directly targeting JAK1 and STAT6 in macrophages. miR-138 targets Sirtuin 1 (SIRT1) in macrophages, thereby activating NF-κB. By targeting Leucine-Rich Repeat-Containing G-Protein Coupled Receptor 4, the miR-34 family (miR-34a, -34c) increases pro-inflammatory cytokines in venous ulcers. miR-132, which is elevated in LPS-stimulated macrophages, increases the production of IL-8 and MCP-1 via SIRT1 regulation and is a biomarker for rheumatoid arthritis and inflammatory bowel disease. By targeting IκBβ, let-7a triggers pro-inflammatory responses that lead to NF-κB activation and the generation of inflammatory and adhesion markers in endothelial cells (57).

However, there is still little direct validation in periodontal tissues, and many of these results come from non-periodontal models (venous ulcers, rheumatoid arthritis, diabetic nephropathy, atherosclerosis).

#### 3.3.1 miRNA regulation of IL-1, IL-6, and TNF-α in periodontal context

By binding to the 3’UTR of target mRNAs or to upstream signaling molecules, miRNAs primarily control the pro-inflammatory cytokines IL-1, IL-6, and TNF-α by preventing translation or promoting transcript degradation (58). Among the miRNAs that have been confirmed in periodontal tissues, miR-155 is a pro-inflammatory mediator that targets SHIP1. This negative immune regulator increases NF-κB activity and stimulates the production of TNF-α and IL-6. It is also linked to M1 macrophage polarization and is consistently elevated in periodontitis (52). On the other hand, miR-146a suppresses NF-κB signaling by targeting TNF Receptor-Associated Factor 6 (TRAF6) and IL-1 Receptor-Associated Kinase 1 (IRAK1). Its persistent increase in periodontitis indicates an inadequate compensatory response to chronic inflammation. TNF-α and IL-6 mRNAs are directly bound by miR-125b, preventing their translation, and their downregulation in periodontitis may contribute to the overproduction of cytokines (39, 59). By targeting the NLRP3 inflammasome and the IL-1β gene, miR-223 suppresses IL-1β signaling; its overexpression in periodontitis is also associated with osteogenic regulation (41, 45, 59).

Furthermore, let-7e and miR-26b directly prevent IL-6 synthesis, whereas miR-378 prevents IL-1β generation by targeting caspase-1. The need for cell-type-specific studies is underscored by the context-dependent effects of miR-21, which are both elevated and downregulated in several periodontitis studies (60, 61).

**Figure 2:**
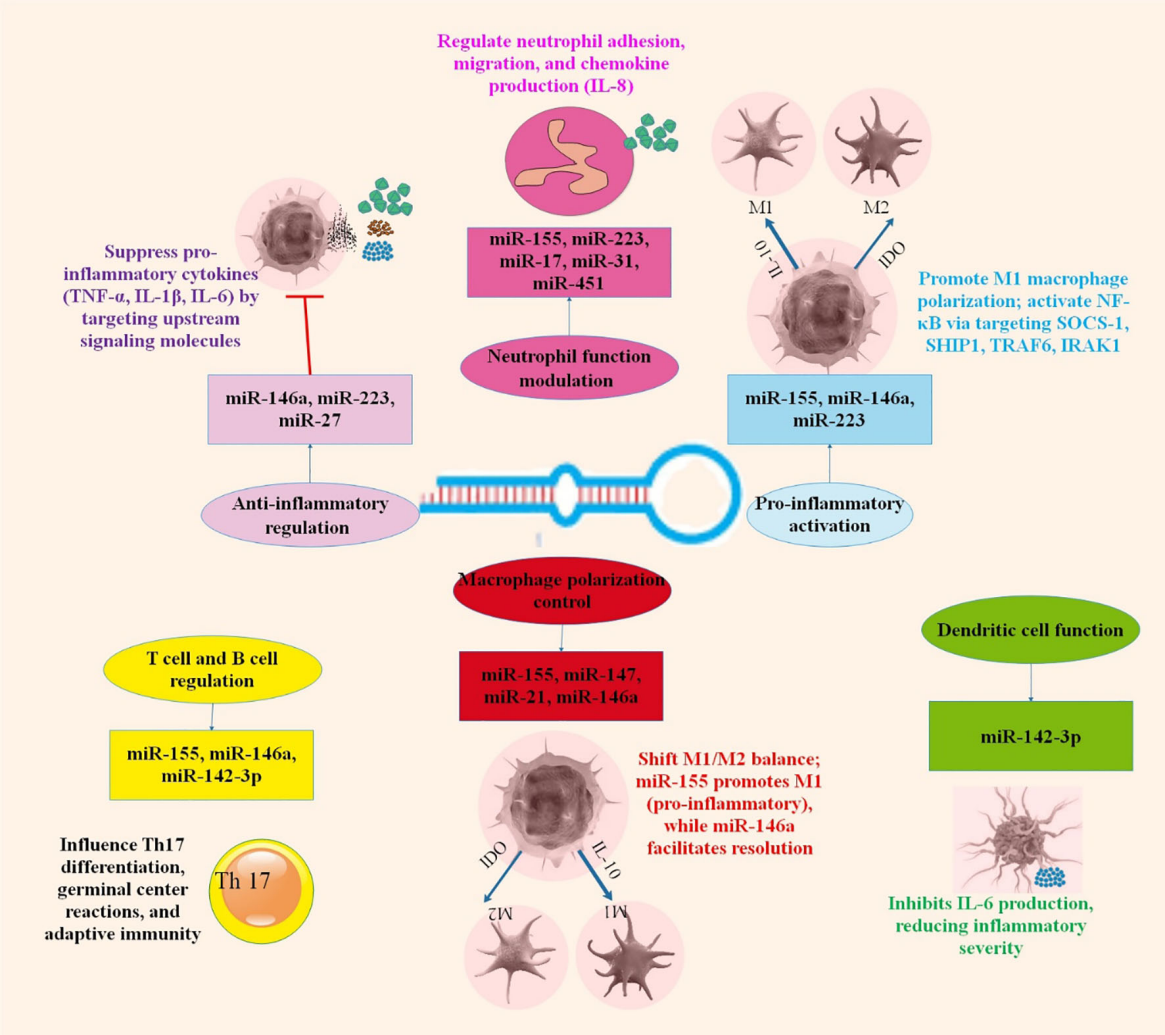
The regulatory functions of miRNAs in host immunological responses during periodontitis. This picture depicts the principal immune cell types and functions influenced by miRNAs in PD. Modulation of neutrophil function: miR-155, miR-223, miR-17, miR-31, and miR-451 govern neutrophil adhesion, movement, and the synthesis of chemokine IL-8. Anti-inflammatory regulation: miR-146a, miR-223, and miR-27 inhibit pro-inflammatory cytokines (TNF-α, IL-1β, IL-6) by targeting upstream signaling molecules including TRAF6, IRAK1, and SHIP1. Macrophage polarization regulation: miR-155 promotes M1 (pro-inflammatory) macrophage polarization, whereas miR-146a and miR-21 promote M2 (resolution) macrophage polarization, thereby altering the M1/M2 equilibrium. Regulation of T cells and B cells: miR-155 and miR-146a affect Th17 development, germinal center responses, and adaptive immunity. miR-142-3p reduces IL-6 synthesis in dendritic cells, thereby reducing inflammation. These miRNA-mediated regulatory networks collectively govern the host immune response, influencing the balance between protective immunity and pathological inflammation in periodontitis.

This intricate regulatory network strictly regulates inflammation. Excessive cytokine release and chronic inflammatory diseases, such as rheumatoid arthritis, sepsis, and inflammatory bowel disease, are caused by dysregulation, such as low miR-146a or high miR-155 (59). Pro-inflammatory (miR-155, miR-21) and anti-inflammatory (miR-146a, miR-125b, miR-223) miRNAs seem to be out of balance in periodontitis, which leads to ongoing tissue damage. The regulating roles of miRNAs in host immune responses during periodontitis are shown in **Figure 2**.

There are several obstacles to the current knowledge of miRNA-mediated cytokine modulation in periodontitis. The context-dependent effects of miRNAs such as miR-155 and miR-21, which exhibit both pro- and anti-inflammatory roles depending on cell type and disease stage, are often overlooked, and many mechanistic insights come from non-PD models (atherosclerosis, diabetic nephropathy, rheumatoid arthritis) without direct validation in periodontal tissues. The majority of research focuses on TNF-α, IL-1, and IL-6, with little attention paid to other inflammatory mediators, including chemokines and IL-17. Additionally, there remains a lack of functional validation (such as miRNA overexpression or deletion in periodontal cells), and cross-sectional designs cannot determine whether miRNA alterations cause or result from inflammation. Functional validation in periodontal cell types, examination of IL-17/Th17-associated miRNA regulation, longitudinal studies to establish temporal causality, and investigation of miRNA-cytokine networks as therapeutic targets using antagomirs or mimics in preclinical models should be the top priorities for future research.

### 3.4 Role of miRNAs in PDL stem cell differentiation and osteogenesis

PDLCs are a heterogeneous population capable of differentiating into fibroblasts, osteoblasts, and cementoblasts. PDLSCs exhibit self-renewal and multidirectional differentiation potential, enabling the formation of cementum-PDL-alveolar bone complexes (62). Several miRNAs regulate osteogenesis in PDL-derived cells and play major roles in bone remodeling during periodontitis (63).

Mesenchymal stem cell (MSC)-derived miRNAs exist in two forms: intracellular miRNAs, which regulate gene expression within the same cell, and exosomal miRNAs, which are released in EXOs and mediate intercellular communication (64). **Table 3** summarizes the regulatory functions of miRNAs across different dental stem cell (DSC) types, underscoring their therapeutic potential for periodontal and pulpal regeneration.

**Table 3:** Overview of miRNA-mediated regulation in DSCs, with implications for periodontal and pulpal regeneration.

Dental stem cell type	miRNA	Study type	Biological effect	Mechanism	Ref
Dental Pulp Stem Cells (DPSCs)	miR-125a-3p	*In vitro*	Reduces inflammation and promotes odontoblastic differentiation	Targets Fyn and suppresses the Toll-Like Receptor (TLR) and NF-κB signaling pathways.	(65, 66)
DPSCs	let-7c-5p	*In vitro* and *in vivo*	Anti-inflammatory; promotes osteogenesis	Suppresses HMGA2/PI3K/Akt signaling during acute pulpitis	(67)
DPSCs	miR-224-5p	*In vitro*	Augments proliferation and migration	The downregulation of miR-224-5p enhances cellular motility and proliferation.	(68)
DPSCs	miR-221-3p	*In vitro*	Modulates cellular viability and programmed cell death	Depletion triggers Rac1-mediated apoptosis.	(69, 70)
PDLSCs	EXO-miR-205-5p	*In vitro* and *in vivo*	Mitigates inflammation; reinstates Th17/Treg equilibrium	Targets X-Box Binding Protein 1 in CP	(71)
PDLSCs	miR-21	*In vitro* and *in vivo*	Modulates osteogenic differentiation	Targets Smad5; silencing disrupts osteogenesis.	(72)
PDLSCs	miR-7	*In vitro*	Preserves stemness and regulates osteogenic differentiation	Regulates Krüppel-Like Factor 4 via Cerebellar Degeneration-Related protein 1 antisense RNA network	(73, 74)
PDLSCs	miR-223	*In vitro* and patient-derived	Impedes osteogenic differentiation and is correlated with the severity of periodontitis	Directly targets FGFR2 and TGFβR2; upregulated in inflamed gingiva	(63)
PDLSCs	miR-508-5p	*In vitro*	Impedes osteogenic differentiation	Specifically targets SOX11; its downregulation enhances osteogenesis.	(75)
PDLSCs	Exosomal miRNAs (72 upregulated, 35 downregulated)	*In vitro*	Promotes BMSC osteogenesis	Involves MAPK, AMPK, and insulin signaling pathways	(76)
PDLSCs (human PDLCs)	miR-299-5p	*In vitro* (co-culture)	Inhibits hMSC osteogenesis while preserving stemness	Regulated by POSTN; targets SOX11	(77)
PDLSCs	miR-584-5p	*In vitro* (bioinformatics + validation)	Inhibits osteogenic differentiation	Identified from Gene Expression Omnibus datasets (GSE99958, GSE159507, GSE159508); possible miRNA-mRNA interaction network	(78)
DFSCs	EXO-miR-140-3p	*In vitro* and *in vivo*	Inhibits PDLC pyroptosis; reduces RR	MiR-140-3p directly targets DNMT1, leading to modified methylation of the SOCS1 promoter. This epigenetic alteration inhibits NF-κB signaling, thereby diminishing NLRP3-dependent pyroptosis.	(11)
Stem Cells from Human Exfoliated Deciduous Teeth (SHED)	miR-26a	*In vitro* and *in vivo*	Promotes angiogenesis in pulp regeneration	Shuttled via SA-EXO; activates TGF-β/SMAD2/3 signaling	(79)
SHED	miR-221	*In vitro*	Induces neuronal differentiation	Binds to CHD8; activates Wnt/β-catenin pathway	(80)
Stem Cells from Apical Papilla (SCAP)	Hsa-let-7b	*In vitro*	Suppresses odonto	Inhibits matrix metalloproteinases (MMP) 1 expression	(81)
SCAP	miR-34a	*In vitro*	encourages the differentiation of odonto/osteogenic	Interacts with the Notch signaling pathway	(82)
SCAP	miR-497-5p	*In vitro*	Osteo/odontogenic differentiation is stimulated.	Activates the Smad signaling pathway by targeting Smurf2	(83)
Dental Follicle Cells (DFCs)	miR-101	*In vitro*	Supports osteogenic differentiation	Increases Alkaline Phosphatase (ALP) activity and osteogenic transcription factors (*e.g.*, SP7)	(84)
DFCs	miR-146a	Patient-derived (Cleidocranial Dysplasia)	Modulates osteogenic gene expression	Regulates Colony Stimulating Factor 1, Runt-Related Transcription Factor 2 (RUNX2), Epidermal Growth Factor Receptor, and OPG; downregulated in RUNX2+/m DFCs	(85)

#### 3.4.1 Key miRNAs regulating PDLSC osteogenesis

Based on their functional effects, many microRNAs (miRNAs) control osteogenic differentiation in PDLSCs. These miRNAs may be classified into three groups: inhibitory, promotive, and context-dependent. On the one hand, miR-132 levels decrease during osteogenesis and hinder bone production via Growth Differentiation Factor 5 (GDF5) targeting and NF-κB activation; on the other hand, miR-31 reduces osteogenesis by lowering Osterix (SP7) expression. In a similar vein, miR-223, which is more prevalent in inflamed gingiva and is associated with the severity of periodontitis, inhibits osteogenesis by directly targeting Fibroblast Growth Factor Receptor 2 (FGFR2) and ansforming Growth Factor Beta Receptor 2 (TGFβR2) (63), miR-508-5p suppresses differentiation by targeting SRY-Box Transcription Factor 11 (SOX11) (75), and miR-584-5p has been recognized as a negative regulator by bioinformatic analysis (78). Promotive microRNAs, in contrast to these inhibitory ones, include miR-589-3p, which targets Activating Transcription Factor 1 (ATF1), and miR-628-5p, which targets ETV1, GATA6, and SOX11, and so promotes MSC development. Nevertheless, miRNAs’ functional roles are not always clear-cut, as shown by miR-21, which displays conflicting effects based on cell type and mechanical context: through the PTEN/PI3K/Akt/Hypoxia-Inducible Factor 1-Alpha pathway, it promotes angiogenesis and osteogenesis in BMSCs, but it inhibits osteogenic differentiation in PDLSCs by targeting Small Mother Against Decapentaplegic (Smad)5, and under mechanical stress, it switches to promoting osteogenesis through ACVR2B targeting (64). The importance of carefully interpreting the impact of miRNA-mediated processes in periodontal regeneration is underscored by the fact that their activity is highly dependent on the surrounding environment and the cell type.

#### 3.4.2 Exosomal miRNAs in PDLSC-mediated regeneration

New functions for PDLSC-derived exosomal miRNAs in cell-to-cell communication and bone regenerative processes are being explored:

Liu et al. (76) showed that rat BMSCs may be stimulated to differentiate into osteogenic cells by increasing the osteogenic capacity of EXOs generated from PDLSCs. Exosomal miRNAs were found to be increased by 72 and downregulated by 35 after osteogenic stimulation, according to RNA sequencing. Analyses of pathways hinted at involvement of insulin signaling, AMP-activated protein kinase, and Mitogen-Activated Protein Kinase (MAPK) (76). Although further human confirmation is required, our results provide preliminary evidence that exosomal miRNAs released by osteogenically differentiated PDLSCs enhance bone formation.

Through the actions of miR-299-5p and SOX11, human PDLCs control Human MSCs (hMSCs) osteogenesis, as shown by Kaneda-Ikeda et al. (77). While SOX11 and miR-299-5p were upregulated in co-culture, osteogenesis was downregulated. Blocking the secreted PD-LCL factor periostin (POSTN) restored osteogenesis and SOX11 expression. On the other hand, both miR-299-5p and SOX11 were upregulated by exogenous POSTN. This finding highlights the importance of POSTN in regulating miR-299-5p and SOX11 in hMSCs, which might have consequences for periodontal regeneration (77).

To reduce root resorption (RR) and prevent PDLC pyroptosis, Li et al. (86) investigated EXOs derived from DFSC. Osteoclast generation, M1 macrophage polarization, and NLRP3-mediated pyroptosis were all inhibited by force-EXOs. One important mediator, miR-140-3p, was shown to target DNA Methyltransferase 1 (DNMT1) and alter SOCS1 DNA methylation, thereby reducing NF-κB activation. Inhibitors of miR-140-3p had the opposite effect. Consequently, exosomal miR-140-3p generated from DFSC activates the DNMT1/SOCS1/NF-κB pathway to prevent PDLC pyroptosis and RR (86). Regenerative periodontal and pulp health are affected by miRNA-mediated control of DSCs, which is discussed in **Table 3**.

Several limitations affect current studies on miRNAs in PDLSCs and EXO-mediated regeneration, beginning with an overreliance on *in vitro* models and insufficient *in vivo* validation in large-animal periodontitis models. Compounding this issue are contradictory findings, most notably that miR-21 promotes osteogenesis in BMSCs but inhibits it in PDLSCs, suggesting that miRNA effects are highly context-dependent and remain poorly understood. Furthermore, most mechanistic validations are incomplete, and critical variables such as the optimal cell source (DFSCs versus PDLSCs), dosage, and delivery method for EXO-based therapies have yet to be determined. The lack of studies examining the inflammatory periodontal microenvironment further complicates translation, as this milieu impairs PDLSC function and may differentially affect PDLC subpopulations. Additionally, the absence of standardized protocols for EXO isolation and miRNA normalization hinders reproducibility across studies. To address these gaps, future research should prioritize large-animal models, single-cell miRNA profiling, rigorous *in vivo* functional validation under inflammatory conditions, and early-phase clinical trials to evaluate EXO-based periodontal regeneration strategies.

**Figure 3:**
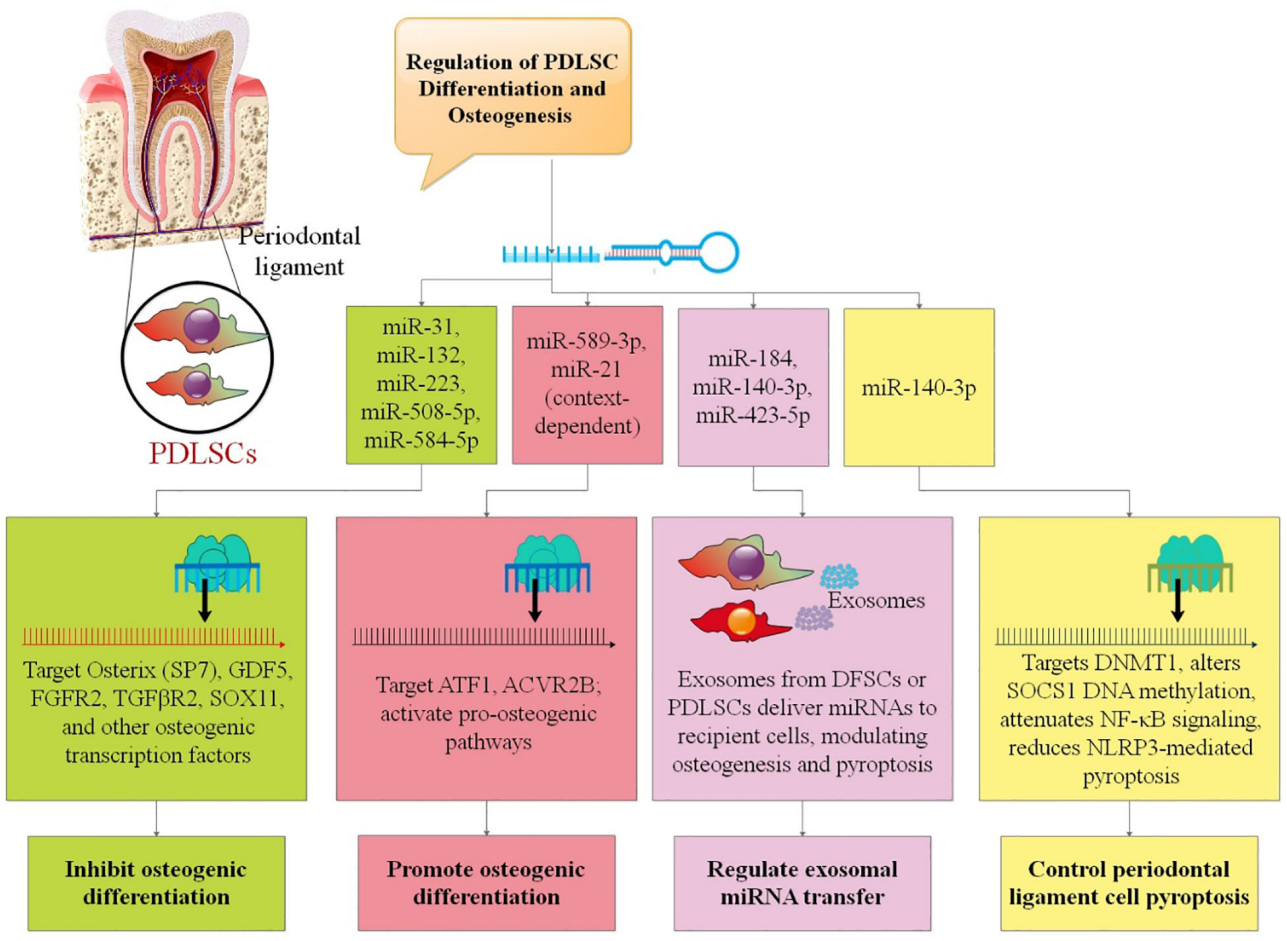
Regulation of PDLSC differentiation, osteogenesis, and exosomal signaling by miRNA. Inhibitory miRNAs (miR-31, miR-132, miR-223, miR-508-5p, miR-584-5p) impede osteogenesis by targeting SP7, GDF5, FGFR2, TGFβR2, and SOX11. Promotive miRNAs (miR-589-3p, miR-21) stimulate bone growth through ATF1 and ACVR2B. Exosomal miRNAs (miR-184, miR-140-3p, miR-423-5p) facilitate intercellular communication, whilst miR-140-3p also regulates pyroptosis in PDLCs.

**Figure 3** illustrates the regulation of PDLSC differentiation, osteogenesis, and exosomal signaling by miRNA.

### 3.5 miRNAs modulating the RANKL/OPG axis in alveolar bone resorption

By modifying the RANKL/OPG axis at the post-transcriptional level, miRNAs play a crucial role in controlling alveolar bone resorption. This affects osteoclast development and activity in both physiological conditions (such as orthodontic tooth movement (OTM)) and pathological states (such as periodontitis and peri-implantitis). A network of miRNAs with conflicting effects carefully tunes this axis: miR-106b reduces osteoclast development by targeting RANKL, whereas pro-resorptive miRNAs (miR-21, miR-3198, and miR-217) accelerate bone loss by targeting OPG and raising the RANKL/OPG ratio (87). Conversely, protective miRNAs (miR-1260b, miR-335, miR-377, miR-26a) suppress resorption by reducing RANKL expression or inhibiting osteoclastogenesis, and miR-503 and miR-144-3p directly target the RANK receptor to block osteoclast activity. Because these miRNAs can be delivered via EXOs derived from MSCs or PDLCs, they hold promise for treating alveolar bone loss in periodontitis, OTM, peri-implantitis, and other osteolytic diseases (88–91).

The RANKL/RANK/OPG system governs alveolar bone metabolism: RANKL binding to RANK accelerates osteoclastogenesis and bone resorption. At the same time, OPG inhibits these processes, with the RANKL/OPG ratio determining the net balance of bone loss and formation. Stem cell-derived EVs (SC-EVs) show promise as cell-free therapies by reducing this ratio, promoting M2 macrophage polarization, and enhancing bone formation. The OPG content of SC-EVs is critical for their efficacy, and EVs from inflammation-preconditioned stem cells confer stronger protective effects. Despite standardization challenges, SC-EVs targeting this pathway show potential for treating alveolar bone defects, accelerating OTM, and preventing periodontitis-induced bone loss (92).

#### 3.5.1 miRNAs in orthodontic tooth movement

Using a split-mouth technique, Polizzi et al. (87) investigated miRNA expression during OTM in 21 healthy teenagers. OTM dramatically altered miRNA expression on the tension and compression sides after three months. While miR-125b-5p, miR-200b-3p, and miR-200b-5p were downregulated on the tension side, miR-7a-2-3p, miR-21-5p, and miR-100-5p were upregulated. Greater OTM at three months was predicted by higher baseline levels of miR-7a-2-3p, miR-21-5p, and miR-200b-3p, as well as lower levels of miR-125b-2-3p, younger age, and less bleeding on probing (87). Although generalizability is limited by the small sample size (n=21) and brief follow-up, our results imply that GCF miRNAs may function as early predictors of individual OTM response.

The functions of miR-214 and miR-206 in OTM were reviewed by Salehivaziri et al. (93), with an emphasis on their role in limiting osteoblast development by targeting EphrinA2. This membrane-bound protein stimulates bone formation via binding to the EphA2 receptor. Osteoblast function and bone remodeling are compromised by dysregulation of the miR-214/miR-206–EphrinA2 pathway, which may slow and reduce OTM stability (93). Nevertheless, the therapeutic significance of this proposed mechanism remains unknown and has not been confirmed *in vivo*.

#### 3.5.2 IL-17A/JAK/STAT3/RANKL axis in periodontitis

By stimulating osteoclasts, Th17 cells, and IL-17A contribute to alveolar bone loss in periodontitis. According to Lv et al. (94), IL-17A improves osteoclast activity by increasing RANKL synthesis in PDLCs via JAK/STAT3 activation. They demonstrated that tofacitinib, a JAK inhibitor, dramatically decreased alveolar bone loss by blocking Th17 differentiation and osteoclast activity using a ligature-induced periodontitis model (94). These results suggest tofacitinib as a possible treatment and highlight the IL-17A/JAK/STAT3/RANKL axis in PDLCs as a primary cause of periodontal bone loss. Nevertheless, the ligature-induced model does not accurately mimic clinical polymicrobial periodontitis, and further research is needed to determine if systemic vs local tofacitinib is safer.

**Figure 4:**
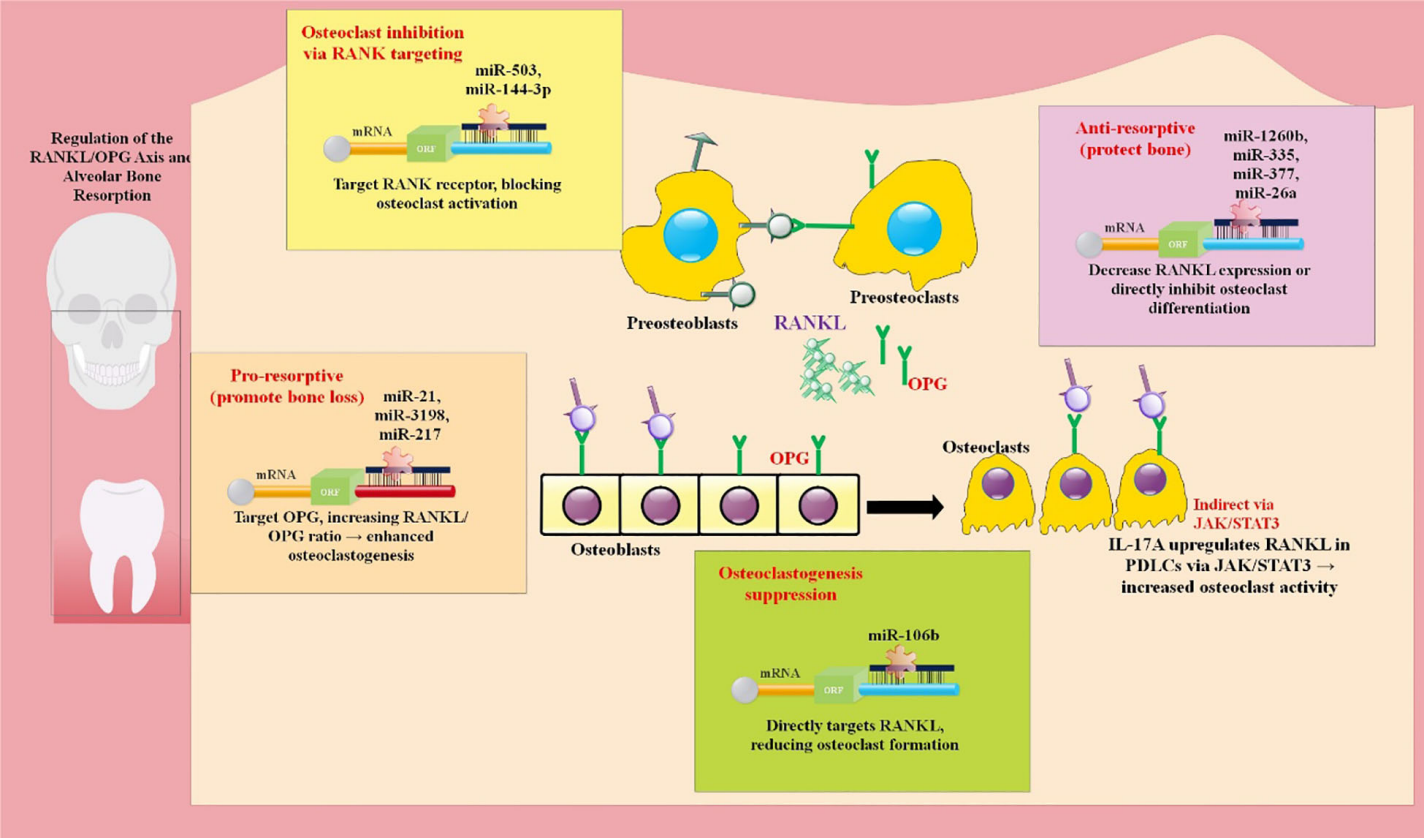
In periodontitis, miRNA regulates the RANKL/OPG axis and alveolar bone resorption. This schematic shows how miRNAs play two roles in osteoclast-mediated bone loss. Pro-resorptive miRNAs cause bone loss: miR-21, miR-3198, and miR-217 target OPG, raising the RANKL/OPG ratio and preosteoblast osteoclastogenesis. Anti-resorptive miRNAs, including miR-1260b, miR-335, miR-377, and miR-26a, limit osteoclast development or RANKL production, maintaining alveolar bone. MiR-503 and miR-144-3p directly target the RANK receptor to suppress osteoclast activity. MiR-106b directly targets RANKL, inhibiting osteoclast production. The Th17/IL-17A pathway: Through the JAK/STAT3 signaling pathway, it increases RANKL expression in PDLCs, which increases osteoclast activity and alveolar bone resorption. These miRNA-mediated regulatory networks balance PD, bone growth, and resorption.

The RANKL/OPG axis and alveolar bone resorption in periodontitis are regulated by miRNA, as shown in **Figure 4**.

Research on miRNA regulation of the RANKL/OPG axis is hampered by several factors, such as a heavy reliance on *in vitro* and small-animal models with little human validation, as demonstrated by the one human OTM study with only 21 participants and a brief follow-up (87), and conflicting miRNA roles, such as the poorly understood dual function of miR-21 as a predictive biomarker and a pro-resorptive signal. Furthermore, the ligature-induced periodontitis model (94) does not accurately mimic polymicrobial disease, and suggested pathways, such as the miR-214/miR-206–EphrinA2 pathway for OTM (93), require *in vivo* validation. Standardizing SC-EV production and dosage remains a major challenge. More research is also needed to determine the safety of systemic vs local tofacitinib. The most promising targets, according to this evaluation, are miR-21, miR-106b, and miR-26a. Future research should focus on large longitudinal human studies, standardized SC-EV protocols, rigorous *in vivo* validation using antagomirs or mimics, combination therapies targeting multiple nodes of the RANKL/OPG axis, and safety/efficacy studies of JAK inhibitors in periodontitis.

### 3.6 Conflicting findings and unresolved questions in miRNA periodontal research

Clinical translation is complicated by contradictory findings, despite strong evidence linking miRNAs to periodontitis. The most significant disparity concerns miR-21, which has been reported to be both upregulated and downregulated in various studies. This is probably because miR-21 plays opposing roles in different cell types, promoting osteogenesis in BMSCs but inhibiting it in PDLSCs, with additional modulation by mechanical forces (64). The most dependable diagnostic possibilities, on the other hand, are miR-146a, miR-155, and miR-223, which show consistent overexpression across many independent cohorts (41–43, 45, 47, 52). Since miRNAs identified using *P. gingivalis* LPS in cell cultures are often undetectable in live infection models (51), a crucial unsolved problem is the poor correlation between *in vitro* and *in vivo* results. This suggests that pure LPS does not reproduce the complexity of bacterial infection. With AUC values ranging from 0.69 to 0.997, diagnostic accuracy also varies widely, likely due to differences in sample types, normalization techniques, and disease criteria. Additionally, several miRNAs have context-dependent functions. For example, miR-155 can promote inflammation in certain contexts while having anti-inflammatory effects in others, and miR-223 can inhibit both inflammation and osteogenesis (57, 63). However, most studies ignore this complexity by reporting only changes in expression without functional validation in specific cell types. Cross-study comparison is made more difficult by methodological variation in sample collection, standardization, and detection techniques. There are also fundamental uncertainties about polymicrobial interactions, cell-type-specific contributions, causation vs correlation, and the therapeutic window for miRNA-based therapies. Standardized procedures, large longitudinal cohorts, single-cell profiling, polymicrobial animal models, functional validation, and independent cohort validation of diagnostic panels should be prioritized in future research to address these issues.

The treatment approaches described in Sections 4–6 are directly influenced by the miRNA-mediated pathways mentioned above, namely the pathogenic overexpression of miR-155, miR-223, and miR-338-3p as well as the protective functions of miR-146a-5p, miR-27, and miR-210. In particular, the most sensible targets for miRNA mimic or anti-miR delivery systems are the same miRNAs that function as diagnostic biomarkers (miR-146a, miR-155, and miR-223) and important regulators of the RANKL/OPG axis and PDLSC osteogenesis. The next section examines how these specific miRNA payloads may be delivered to diseased periodontal tissues via sophisticated delivery systems (NPs, hydrogels, EXOs).

### 3.7 miRNA regulation of matrix metalloproteinases and tissue inhibitors of metalloproteinases

A crucial difference in understanding the pathophysiology of periodontitis is that MMPs and TIMPs form a distinct proteolytic pathway that drives connective tissue breakdown, in addition to the RANKL/OPG axis, which controls bone resorption. MiRNAs have emerged as upstream regulators of this network, and MMPs degrade extracellular matrix (ECM) components, especially collagens, whereas TIMPs regulate this activity (95, 96).

About 80% of the overall collagenase activity in GCF is attributed to neutrophil-derived MMP-8 (collagenase-2). The severity of the condition and the effectiveness of treatment are consistently correlated with elevated GCF and salivary levels of MMP-8, -9, -13, -3, -7, -25, and -26. Chairside MMP-8 testing enables real-time monitoring, and a persistent increase in MMP-8 levels indicates poor treatment outcomes. Early implant failure has been linked to genetic variations in MMP-8 and MMP-1, indicating that MMP genotyping might improve patient selection (97, 98).

#### 3.7.1 miRNAs directly targeting MMPs

By specifically targeting Mmp9, miR-218 provides protective benefits. MMP-9 is increased, and miR-218 is downregulated in rat periodontium and human PDLCs treated with LPS. While Mmp9 overexpression has the opposite effects, miR-218 overexpression lowers inflammatory cytokines, inhibits osteoclast formation, and decreases collagen breakdown. In a rat model of periodontitis, miR-218 reduces inflammation and bone resorption *in vivo*, making the miR-218/Mmp9 axis a potential therapeutic target (99).

In human PDLCs, miR-146a reduces LPS-induced elevations in MMP-2 and MMP-9 (100–102).

Furthermore, MMP-9 activity in GCF has been linked to miR-146a and miR-155; both miRNAs decrease after non-surgical periodontal treatment, and their overexpression correlates with increased MMP-9 levels in individuals with periodontitis (99, 103). Additionally, MMP-2 and MMP-9 expression in apical periodontitis has been shown to be regulated by miR-125b and miR-29b, indicating common regulatory mechanisms across periodontal disorders (104).

Salivary miR-146a was significantly elevated in periodontitis, along with elevated inflammatory cytokine levels and clinical indicators, in a study comparing 50 healthy controls with 68 patients with CP. The degree of gingival inflammation and MMP-8/TIMP-1 levels were positively associated with miR-146a, indicating that salivary miR-146a might serve as a noninvasive biomarker for tracking periodontal inflammation (105).

#### 3.7.2 Indirect miRNA-mediated MMP regulation

By inhibiting SOCS3 and SOCS5, important negative regulators of JAK/STAT signaling, in TLR4-stimulated macrophages, miR-451a is an example of indirect miRNA-mediated MMP control (106). This suppression promotes M1-like macrophage polarization, increases NF-κB activation, alleviates JAK/STAT inhibition, and increases MMP-8 and MMP-9 synthesis. MiR-451a expression is negatively associated with M2 markers in inflammatory gingival tissues, suggesting its involvement in the maintenance of tissue degradation. Therefore, miR-451a indirectly regulates MMPs and TIMPs, linking bacterial challenge, immunological reprogramming, and proteolytic destruction (105, 107).

#### 3.7.3 Exosomal miRNAs and TIMP regulation

By altering the MMP/TIMP balance, exosomal miRNAs play a crucial role in controlling periodontal tissue remodeling. By regulating MMP expression and activity, they influence ECM breakdown; specific miRNAs, such as miR-146a, miR-155, and miR-223, control inflammatory and tissue-destructive pathways. Exosomal miRNAs function as molecular switches that regulate the net proteolytic outcome, alternating between tissue homeostasis and destruction by regulating MMP-mediated collagen breakdown and TIMP-mediated inhibition. Exosomal miRNAs are positioned by this regulatory axis as potential therapeutic targets for regulating MMP activity and repairing the integrity of periodontal tissue (104). In terms of TIMP regulation, ECM turnover is affected by miR-203’s modulation of TIMP-3. Whether miRNA-mediated MMP/TIMP dysregulation precedes or follows increases in inflammatory cytokines is a crucial unanswered question that requires long-term research to establish causation.

The majority of research on miRNA-mediated MMP/TIMP regulation in periodontitis comes from animal or *in vitro* models with little human validation; cross-sectional studies cannot prove a causal relationship between miRNA alterations and MMP dysregulation; many miRNAs target multiple MMPs with poorly defined off-target effects; EXO research lacks standardized isolation protocols; and miRNA-based diagnostics are still experimental despite chair-side MMP tests. Longitudinal studies to establish temporal causality, functional validation in human periodontal tissues, exploration of combined miRNA/MMP-targeting strategies, and clinical trials of treatments targeting both the RANKL/OPG and MMP/TIMP pathways for all-encompassing periodontal tissue protection should be the top priorities for future research.

### 3.8 Methodological quality assessment of current evidence for miRNA regulatory networks in periodontitis

The strengths of the current evidence encompass the consistent upregulation of miR-146a, miR-155, and miR-223 across various independent cohorts and sample types (Guo et al.;^39^–^42^, ^44^, ^45^, ^47^), functional validation of selected miRNAs (miR-184, miR-223, miR-508-5p) via luciferase assays and rescue experiments (44, 63, 86, 104, 105), cross-species validation of several miRNAs (miR-200b, miR-146a, miR-155) between murine models and human tissues (51, 54), and notable diagnostic potential with AUC values surpassing 0.80 for miR-486-5p and the miR-200 family (Guo et al.;^36^).

Small sample sizes (usually 11–50 patients per group), the absence of independent validation cohorts, cross-sectional designs that prevent causal inference, inconsistent normalization techniques (U6 *vs.* cel-miR-39 *vs.* global mean), heterogeneous sample types (tissue, saliva, GCF, plasma), and insufficient control for confounding variables like smoking, diabetes, and medications are some of the major flaws that restrict interpretability (108, 109). Another issue is publication bias that favors favorable results.

Critical gaps are revealed by cross-model translation: some mouse miRNAs (miR-200b, miR-146a, and miR-155) have been confirmed in human tissues, but machine learning-derived signatures lack external human validation. *In vitro* results (LPS-stimulated cells) consistently show miRNA-mediated regulation of MMPs/TIMPs but frequently fail to translate to live infection models (51, 54). The majority of studies are at high risk of bias due to selection bias, insufficient blinding, and poor control of confounding.

Overall quality rating: sample size, validation, research design, and control for confounding are low to moderate; replication of important miRNAs is good. To improve causal inference and speed up clinical translation, future research should focus on large longitudinal cohorts (≥100 patients per group), independent validation cohorts, standardized normalization protocols, strict confounding control, laboratory staff blinding, and compliance with Minimum Information for Publication of Quantitative Real-Time PCR Experiments (for qPCR), Strengthening the reporting of observational studies in epidemiology, standards for reporting of diagnostic accuracy studies, or Transparent Reporting of a Multivariable Prediction Model for Individual Prognosis or Diagnosis reporting guidelines (110, 111).

## 4 Therapeutic inhibition of pathogenic miRNAs (anti-miRs) in periodontal disease

MiRNAs play key roles in regulating innate and adaptive immune responses in periodontitis, affecting T cells, B cells, neutrophils, macrophages, and dendritic cells. Upregulated miRNAs include miR-146a, miR-29, miR-15, miR-148, and miR-223, while miR-31, miR-92, and miR-451 are downregulated. Using RNA mimics and antagomirs to target miRNA pathways represents a promising therapeutic strategy for PD (8). RNA mimics and antagomirs are intriguing treatment approaches, and the stability of miRNAs in GCF supports their use as non-invasive diagnostic indicators (8, 8).

Several miRNAs, such as miR-142-3p, miR-146a, miR-155, miR-203, miR-223, miR-21-3p, and miR-200, have been suggested as biomarkers of PD. For example, miR-146 and miR-155 regulate immune responses and chronic inflammation via cytokines such as RANKL, IL-1, and TNF-α. GCF and saliva are the best sites for collecting samples without causing harm. Over 600 miRNAs have been found in GCF, with higher levels of miR-223 in people with periodontitis (8).

New research has found different miRNA signatures for different pathogens. For example, *Fusobacterium nucleatum* changes miR-205, miR-210, and miR-199a-3p; *S. gordonii* changes miR-375, miR-34b-5p, miR-210, and miR-423-5p; *T. forsythia* lowers miR-375 and miR-200c; *Treponema denticola* changes miR-486, miR-126-3p, and miR-126-5p; and polybacterial infection (PAHMM) involves miR-9, miR-148a, and miR-690 in the pathophysiology of periodontitis (8).

The gingival tissues of individuals with periodontitis and of ligature-induced animal models showed higher levels of the mir338 cluster, which has previously been shown to be a target in osteoporosis (112). Seven days after ligature implantation, Zhang et al. showed that Mir338 cluster knockout mice showed less mandibular bone loss. Mechanistically, ablation of the Mir338 cluster inhibited osteoclastogenesis via Stat1, shifted macrophages toward the M1 phenotype, and reduced osteoclast formation while increasing IFN-γ and JAK-STAT signaling. In periodontitis models, local delivery of miR-338-3p antagomir stopped buccal bone loss. According to our results, the Mir338 cluster provides a unique therapeutic target for halting alveolar bone loss as it regulates M1 macrophage polarization and osteoclastogenesis. Human validation is necessary since the ligature-induced model does not accurately mimic human polymicrobial periodontitis (112).

This study explored how isodrimeninol, a natural sesquiterpene from Drimys winteri, affected six miRNAs related to periodontitis using hPDL-MSCs and LPS-stimulated Saos-2 osteoblast-like cells. Isodrimeninol reduced IL-1β and IL-6 gene expression and altered miRNA levels in different ways. It increased the levels of miRNAs that defend cells (miR-146a-5p and miR-223-3p) and decreased the levels of miRNAs that cause disease (miR-17-3p, miR-21-3p, miR-21-5p, and miR-155-5p). These results show a link between miRNA control and isodrimeninol’s anti-inflammatory effects in periodontitis models. This suggests that isodrimeninol could be used as a therapy, but further research is needed to confirm this (113).

### 4.1 miRNA mimics for restoring protective miRNAs and regenerating bone

Since Victor Ambros and Gary Ruvkun’s discovery of microRNAs in 1993—for which they shared the 2024 Nobel Prize in Physiology or Medicine—miRNA-based therapies have been a primary focus of therapeutic research. The therapeutic goal is either to restore disease-suppressor miRNAs using exogenous mimics or to inhibit pathogenic miRNAs using anti-miRs. Anti-miRNAs are sequence-specific, long-lasting inhibitors first validated against miR-122, and they function effectively with miRNA sponges to sequester overexpressed miRNAs. Successful miRNA delivery requires protection from nuclease degradation, efficient cellular uptake, avoidance of immunotoxicity, such as TLR activation, and biocompatible carriers, as free miRNAs are negatively charged, rapidly degraded, and potentially immunogenic or neurotoxic (114).

As an alternative to bone grafting, scaffold-based bone tissue engineering has become increasingly popular. It addresses problems with allografts by leveraging biological factors, such as gene expression, alongside physical properties (115, 116). Nanofibrous (NF) scaffolds have become popular because they have a high surface-to-volume ratio, are porous, and can imitate the ECM. This helps cells stick together, move, and turn into osteoblasts in BMSCs. NF scaffolds and miRNA mimics work together to create a platform that connects scaffold structure to the stimulation of osteogenic markers. For example, incorporating miRNA-22 and miRNA-126 copies into Polycaprolactone (PCL) nanofibers enhances the survival and osteogenic differentiation of human Induced Pluripotent Stem Cells (iPSCs) by elevating levels of key osteoblast markers, including RUNX2, Bone Gamma-Carboxyglutamate Protein (BGLAP), ALPL, and SPARK. Combining miRNA copies with NF scaffolds is a promising cross-disciplinary approach for bone tissue engineering, but major challenges remain (116).

Angiogenesis is important for bone healing, and miR-210 and miR-16 work in opposite ways. miR-210 turns on both angiogenesis and osteogenesis by blocking Ephrin A3 (EFNA3) and Activin A Receptor Type 1B (AcvR1b), while miR-16 blocks both processes by blocking Vascular Endothelial Growth Factor (VEGF) and Smad5. Castaño et al. (117) created a dual-delivery system that synchronizes angiogenesis and osteogenesis by loading a collagen-nanohydroxyapatite scaffold with a miR-210 mimic and a miR-16 inhibitor. Within 10–14 days *in vitro*, this therapy enhanced the amount of calcium deposited by human MSCs. The first effective dual-miRNA scaffold for bone regeneration was demonstrated at 4 weeks in a rat calvarial injury model, in which dual-miRNA scaffolds produced more than twice the bone volume and 2.3 times the blood vessel recruitment compared to miRNA-free scaffolds (117).

The studies examined for miRNA-based scaffold treatments for bone and gum regeneration are limited because they used only small-animal models (rats), had short follow-up periods, and did not test the therapies in humans. Some important gaps include potential side effects, the ability of delivery vehicles to elicit immune responses, poorly controlled release rates, and the lack of comparisons with gold standards such as BMP-2 or autografts. It is still not well understood how stable miRNA treatments are during storage and in swollen periodontal tissues. Large-animal models, cell-specific delivery systems, long-term safety studies, standardized manufacturing processes, and early-phase clinical trials will all be needed for future studies.

### 4.2 Nanoparticle and hydrogel-based delivery systems for miRNA therapeutics

Building on the mechanistic identification of specific therapeutic miRNA targets, such as osteogenesis-inhibiting miR-223 (Section 3.4), pro-resorptive miR-338-3p (Section 3.5), and inflammation-promoting miR-155 (Section 3.3), efficient clinical translation requires delivery systems that protect these unstable RNA molecules and direct them to specific periodontal cell types. The exact miRNA mimics and anti-miRs mentioned in previous sections, such as miR-146a-5p (anti-inflammatory), miR-27 (pro-osteogenic/angiogenic), and miR-210 (pro-angiogenic), have been explicitly assessed for delivery using the NP, hydrogel, and EXO platforms covered below.

Advancements in drug delivery systems have enabled the creation of customized platforms, such as EVs, lipid-based NPs (LNPs), metallic NPs, and polymer NPs, that enable precise, targeted delivery with fewer systemic side effects (7).

Hydrogels are particularly well-suited for addressing oral and maxillofacial conditions, given the intricate, moist, and dynamic oral environment that renders traditional drug-delivery systems ineffective. Hydrogels, as three-dimensional polymer networks with adjustable features such as swelling, degradability, and stimulus responsiveness, emulate the ECM, thereby ensuring biocompatibility and prolonged drug release. They have been effectively utilized to administer medications, cytokines, and stem cells for antibacterial, anticancer, and tissue-regenerative purposes in disorders such as caries, periodontitis, oral cancer, and mucosal diseases, establishing them as a formidable platform for enhancing oral health (118).

Hydrogels offer several benefits for miRNA distribution, including precise, stimulus-responsive regulation, sustained local release, and prevention of degradation of exogenous miRNA inhibitors or mimics. Hydrogels are more stable, biocompatible, and ECM-like than liposomes and viral vectors (119).

However, because of their short half-life, limited cellular absorption, and off-target effects (“too many targets for miRNA effect”), miRNA-based medications have not progressed beyond phase II studies. MiRNA cargo may be protected and released gradually over days to weeks by hydrogels composed of synthetic (polyethylene glycol, polyvinyl alcohol), natural (alginate, chitosan), or peptide-based materials (120, 121).

To treat irregular oral-maxillofacial bone abnormalities in inflammatory conditions, Yang et al. (122) created Magnesium Silicate Nanospheres (MSNs) loaded with miR-146a-5p (MSN+miR-146a). By targeting TRAF6 and blocking NF-κB, MSN+miR-146a decreased inflammation in LPS-stimulated bone marrow-derived macrophages, enhanced M2 macrophage polarization, and prevented osteoclast formation in human DPSCs (hDPSCs). Using a photocuring hydrogel, these combined osteogenic and immunomodulatory effects were verified in a model of an infected mandibular bone defect (122). This work converted mechanistic knowledge into a therapeutic platform, as anticipated, given the mechanistic function of miR-146a in targeting TRAF6 and suppressing NF-κB activation (39, 59).

Ding et al. (123) studied the use of miR-27 mimic administered by LNPs to target the Wnt signaling inhibitor Secreted Frizzled Related Protein 1 (SFRP1) to improve periodontal tissue repair. In periodontitis models, inflammation reduced miR-27 expression; however, miR-27 therapy increased angiogenic factors (CD31, CD34, VEGF) and osteogenic markers (ALP, RUNX2, Collagen Type I (COL1)), leading to improved vascular sprouting and increased mineral accumulation. *In vivo*, miR-27 release decreased alveolar bone loss distance by 43.9%, produced six times more new blood vessels, and sped up ECM remodeling. By blocking SFRP1, miR-27 mechanistically triggered the Wnt pathway (123). Ding et al. demonstrated that miR-27 mimic administered by LNPs boosted ALP, RUNX2, and COL1 while decreasing alveolar bone loss distance by 43.9%, which is consistent with the osteogenic deficiencies brought on by low miR-27 expression in inflamed periodontal tissues (57, 123).

Li et al. (124) investigated the possibility of preventing alveolar bone loss using EXOs made from human umbilical cord MSCs (hucMSCs-EXO) administered via a hydrogel platform (exo@H). When coupled with hydrogel, lyophilized hucMSCs-EXO improved the proliferation and osteogenic differentiation of MC3T3-E1 cells. Compared with hydrogel alone, exo@H dramatically reduced osteoclast counts, gingival inflammation, and alveolar bone loss in a mouse model of ligature-induced periodontitis. 59 elevated miRNAs were found by miRNA sequencing, including let-7f-5p and miR-203-3p, which target Nit2 and IL-13, respectively (124). In a mouse ligature model, the EXO-based hydrogel platform (exo@H) dramatically decreased osteoclast numbers and alveolar bone loss by delivering let-7f-5p and miR-203-3p, miRNAs that were shown to be differently expressed in periodontitis patients in Section 3.1 (40, 42, 124).

In a pilot investigation, Jalaluddin et al. (125) investigated whether human PDLC migration and proliferation might be enhanced by mRNA expressing Fibroblast Growth Factor 2 (FGF-2) encapsulated in LNPs as opposed to recombinant human FGF-2 (rhFGF-2) protein. After 72 hours, mRNA-FGF2 considerably boosted cell proliferation more than rhFGF-2, suggesting extended protein synthesis. The mRNA group showed a 4.5-fold increase in POSTN expression and a quicker rate of wound closure (88.4% *vs.* 76.1% after 24 hours). The scientists concluded that, as a viable strategy for periodontal regeneration, mRNA technology offers benefits over recombinant protein techniques by enabling continuous protein production (125) (**Table 4**).

**Table 4:** Delivery mechanisms for miRNA-based therapies in periodontal regeneration.

Delivery system	Carrier material	miRNA/Agent loaded	Key features	Outcome	Ref
NF scaffold	PCL nanofibers	miR-22 mimic, miR-126 mimic	High surface-to-volume ratio; acts like ECM; helps cells stick together	Improves iPSCs’ ability to survive and differentiate into bone cells; increases RUNX2, BGLAP, ALPL, SPARK	(115, 116)
Dual-miRNA scaffold	Collagen-nanohydroxyapatite	miR-210 mimic + miR-16 inhibitor	Dual-delivery method; connects bone growth and blood vessel growth	Increase Calcium deposition (10–14 days); 2× bone volume; 2.3× blood vessels	(117)
Nanospheres	Magnesium silicate (MSNs)	miR-146a-5p	Dual effects on bone growth and the immune system control	Encourages hDPSC osteogenesis and M2 polarization while stopping the formation of osteoclasts	(122)
LNPs	LNPs	miR-27 mimic	Targets SFRP1; activates Wnt signaling	increases ALP, RUNX2, COL1, CD31, CD34, VEGF; 6× new blood vessels; 43.9% reduction in bone loss distance	(123)
Hydrogel platform	Photocuring hydrogel	MSN+miR-146a complex	Stimulus-responsive; sustained local release	Confirmed efficacy in the infected mouse mandibular bone defect model	(122)
EXO-based hydrogel	EXOs from hucMSCs + hydrogel (exo@H)	let-7f-5p, miR-203-3p	Lyophilized EXOs were mixed into a hydrogel	Less loss of dental bone, periodontal inflammation, and osteoclast count	(124)
mRNA-LNP	LNPs	mRNA encoding FGF-2	Continuous protein production; superior to recombinant protein	Increase Cell proliferation (72h); faster wound closure (88.4% vs 76.1%); 4.5× POSTN expression	(125)

MiRNA-, EXO-, and mRNA-based periodontal treatments have several drawbacks despite encouraging preclinical findings. The majority of research uses rat or mouse models with just 4–8 weeks of follow-up, which are not very good at simulating long-term healing stability or CP in humans. Biodistribution and potential systemic toxicity of LNPs and MSNs remain poorly understood, and standardization issues with miRNA dosing, delivery methods, and off-target evaluation persist. Uncertain cellular absorption mechanisms, limited native miRNA cargo, inconsistent separation techniques, and a lack of information on the synergistic *vs.* additive effects of dual-miRNA approaches all impede EXO research. Further characterization of hydrogel breakdown rates and release profiles is necessary, and production must comply with Good Manufacturing Practice (GMP) regulations. Furthermore, long-term safety studies (6–12 months) are required to evaluate Wnt pathway activation and immunogenicity, and no head-to-head comparisons with existing clinical standards (Emdogain, GTR membranes, scaling/root planing) have been carried out. In the end, precise, localized delivery techniques will be needed to overcome the basic problem of miRNA off-target effects for clinical translation.

### 4.3 Modulating host immune response via miRNA-targeted therapy

In periodontitis, the inflammatory response is started by bacterial biofilm, but it’s not the only reason. The dysbiosis that causes inflammation remains incompletely understood, and genetic, environmental, and systemic factors all contribute. Standard treatment (biofilm removal) does not consistently restore immune homeostasis, thereby driving interest in host-modulatory drug-based approaches (126). PD occurs when the body’s immune system responds to bacterial infections in the mouth, leading to inflammation and damage. A very important part of controlling immune and inflammatory responses involves miRNAs. miRNAs affect both innate and adaptive defenses in gum disease. They change how T and B cells, neutrophils, macrophages, and dendritic cells work (127).

Through cell-specific pathways in neutrophils, macrophages, gingival fibroblasts, and dendritic cells, miRNAs control innate immune responses in periodontitis (8, 128). MiR-181a suppresses inflammation by targeting IL-8, miR-17 decreases IL-8 via SHIP1, and miR-155 increases IL-8 expression in neutrophils; miR-451 decreases neutrophil migration and inhibits TNF-α and IL-1β production, miR-203 worsens disease by downregulating SOCS3, and miR-126 promotes chemokine synthesis for neutrophil recruitment (8, 128). By targeting IRAK1 and TRAF6 to lower NF-κB activity, miR-146a reduces pro-inflammatory cytokines (IL-6, TNF-α, IL-8, and IL-1β) in gingival fibroblasts; miR-126 similarly targets TRAF6. Key inflammatory regulators in macrophages are miR-146a and miR-155; *P. gingivalis* stimulates miR-132 and miR-21; miR-21 reduces inflammation by targeting Programmed Cell Death 4 but suppresses M2 polarization through STAT3, while miR-147 promotes M1 polarization and miR-214 prevents PDLSC osteogenic differentiation by targeting Catenin Beta 1 or ATF4 (128, 129). MiR-142-3p suppresses IL-6 expression in dendritic cells, thereby reducing the intensity of inflammation. Notably, miR-155 demonstrates context-dependent functions, increasing TLR4/NF-κB activation by suppressing SOCS-1 and SHIP1 or reducing signaling by downregulating MyD88 and IκB Kinase, and suppressing osteoclastogenesis by targeting SOCS-1 and Microphthalmia-Associated Transcription Factor; additionally, miR-142 increases in response to TNF-α in gingival epithelial cells (GECs), while miR-17 is linked to decreased expression of IL-8 (128, 130, 131).

A potential strategy to recalibrate abnormal inflammation without unduly impairing antimicrobial defenses is to target miRNAs to modify host immune responses (132). Pathogenic miRNAs that are overexpressed in periodontitis, such as miR-155, miR-146a, miR-21, and miR-17-3p, encourage the polarization of M1 macrophages, the development of osteoclasts, and the prolonged production of TNF-α, IL-1, and RANKL. On the other hand, protective miRNAs (miR-146a-5p, miR-223-3p, and miR-27) are often downregulated, thereby hindering the resolution of inflammation (133).

By inhibiting pathogenic miRNAs, anti-miR tools (anti-miR-217, anti-miR-155, and anti-miR-338-3p) may reduce M1 skewing, restore efferocytosis, and lower osteoclast activity. Restoring protective miRNAs, blocking NF-κB signaling, and encouraging the resolution of inflammation are all possible using miRNA mimics (miR-146a, miR-223, and miR-27). EXOs, hydrogels, and LNPs are examples of advanced delivery methods that allow for targeted, prolonged, and stimulus-responsive release with few adverse effects. This strategy shifts the host immune profile from a chronic inflammatory state to a pro-resolving, regenerative phenotype (133) (**Table 5**).

**Table 5:** Therapeutic approaches aimed at miRNAs in periodontal disease.

Therapeutic strategy	Agent type	Target miRNA	Mechanism of action	Preclinical effect	REF(s)
Anti-miR therapy	Antagomir/Anti-miR	miR-338-3p	Blocks the Mir338 cluster and uses Stat1 to prevent M1 macrophage polarization and osteoclastogenesis.	Minimizes the loss of mandibular bone and stops the resorption of alveolar bone	(112)
Anti-miR therapy	Anti-miR	miR-155	Decreases M1 skewing and suppresses SOCS-1 and SHIP1	Reduces pro-inflammatory cytokines (IL-1, TNF-α, and RANKL).	(132, 133)
Anti-miR therapy	Anti-miR	miR-217	Suppresses osteoclast activity and restores efferocytosis	Prevents the loss of alveolar bone	(133)
miRNA mimic therapy	miRNA mimic	miR-146a-5p	Targets TRAF6; inhibits the NF-κB pathway	Decreases inflammation and increases M2 polarization	(113, 122)
miRNA mimic therapy	miRNA mimic	miR-223-3p	Suppresses pro-inflammatory cytokine production	Reduces inflammation and raises protective miRNA levels.	(113, 133)
miRNA mimic therapy	miRNA mimic	miR-27	Targets SFRP1; activates Wnt/β-catenin pathway	Encourages angiogenesis and osteogenesis; reduces the distance of bone loss by 43.9%.	(123)
miRNA mimic therapy	miRNA mimic	miR-210	Promotes angiogenesis and osteogenesis while blocking EFNA3 and AcvR1b.	Enhances bone volume and improves bone repair	(117)
miRNA mimic therapy	miRNA mimic	miR-16 inhibitor (combined with miR-210 mimic)	Blocks VEGF and Smad5	Dual delivery: 2.3× blood vessel recruitment and 2× bone volume	(117)
Natural compound	Isodrimeninol (sesquiterpene)	Increase miR-146a-5p, miR-223-3p; decrease miR-17-3p, miR-21-3p, miR-21-5p, miR-155-5p	Modulates miRNA expression; lowers IL-1β and IL-6 production	In models of periodontitis, anti-inflammatory actions	(113)

MiRNA-based immunomodulation for periodontitis has some drawbacks despite promising discoveries. The majority of data come from tiny rodent models or *in vitro* models without human clinical validation, and miRNAs such as miR-155 exhibit inconsistent effects across different cell types, suggesting that cell-specific mapping is incomplete. Cross-study comparisons are complicated by methodological inconsistencies, different isolation techniques, internal controls, sample sizes, and observation times; off-target effects, biodistribution, and environmental/genetic influences (*e.g.*, smoking, diabetes, host genomics) remain poorly understood. Large-animal models, long-term safety studies (6–12 months), cell-type-specific delivery systems, genome-wide off-target analyses, standardized dosing protocols, GMP-compliant production, and direct comparisons with gold-standard therapies (scaling/root planing, antibiotics, Emdogain) should be the top priorities for future research. Clinical translation won’t happen until these problems are addressed.

## 5 Challenges in miRNA-based therapy: specificity, stability, and off-target effects

Therapeutics targeting miRNAs showed significant potential for treating illnesses associated with abnormal gene expression. The creation of miRNA-based drugs, on the other hand, still faces many challenges, such as ensuring stability, targeting specific cells, penetrating tissues, and triggering defensive responses. The therapeutic miRNA drugs have not yet been used in clinical practice, but some are now in clinical trials (128).

Three interrelated issues have made miRNA treatments more difficult than early laboratory research indicated (134): 1. Stability and delivery: When administered, unmodified miRNAs are quickly broken down by ubiquitous nucleases. They are physiologically inactive, even if they escape degradation, because they have difficulty crossing cell membranes and often become trapped in endosomes (135). 2. Off-target effects: A single miRNA may naturally control dozens to hundreds of genes via incomplete seed-region matching. Unintended targets may be silenced by high dosages of mimics or antagomirs, which may have unforeseen or dangerous adverse effects (135). 3. Patient variability: Smoking, diabetes, the makeup of the oral microbiota, and genetic susceptibility all have a substantial impact on the miRNA profiles of individuals with periodontitis. A miR-146a mimic that works for one patient may not work for another, or may even be harmful (136). Researchers have sought to circumvent these challenges by creating chemically modified RNAs that are resistant to degradation and by encapsulating them in delivery systems such as hydrogels, LNPs, or EXOs. However, even these carriers can occasionally provoke undesirable immune responses or localize in inappropriate organs. Until researchers develop improved methods to maintain miRNA stability, mitigate off-target silencing, and account for patient variability, these treatments will remain theoretically promising yet challenging to use in routine dental practice (136).

The three primary problems remain unresolved: interpatient variability, extensive target networks that lead to off-target effects, rapid nuclease degradation, and a negative charge that impedes cellular absorption (33, 137). No miRNA-based medication has advanced beyond Phase II clinical trials for any disease, despite chemical modifications and sophisticated delivery methods. MiRNA treatments for periodontitis will remain preclinical until better techniques are developed to preserve stability, reduce off-target silencing, and account for patient variability (2024; 128).

## 6 Personalized periodontal medicine: leveraging miRNA signatures for tailored treatment

By combining genomic profiling with conventional diagnostics to develop innovative methods for disease diagnosis and treatment, precision medicine seeks to select the best course of action based on each patient’s distinct genetic, environmental, and clinical profile (138, 139). In line with the biopsychosocial model of health, this method goes beyond biological data to include psychological, socioeconomic, and environmental aspects. It also acknowledges the importance of the exposome and external stresses in the presentation of illness (138, 140, 141).

One of the main causes of tooth loss, periodontitis, is both an infectious disease and a noncommunicable condition strongly associated with lifestyle factors. It is characterized by chronic inflammation that is influenced by environmental and host factors (142). PD management varies according to each patient’s specific circumstances. Despite established treatment recommendations, optimization remains challenging due to the extensive array of treatment alternatives and the diverse expertise and perspectives of the practitioners involved. The contemporary medical paradigms of “precision medicine” and “personalized medicine” provide enhanced predictive therapy compared to traditional approaches by meticulously stratifying patients and tailoring treatment modalities accordingly (21). This necessitates a novel diagnostic approach that amalgamates data on unique patient backgrounds (biomarkers, genetics, environment, and lifestyle) with traditional medical examination information. Presently, several biomarkers and novel diagnostic indices are under investigation, alongside studies of PD-associated genes and the intricacies of the oral microbiota (143). In PD, advancements in biomarkers, genetic analysis, and microbiome research are establishing the foundation for precision strategies, presenting the possibility of more effective, personalized treatment. The progression of PD is complex, varying by tooth and influenced by biofilm and occlusal stress, rendering therapy inherently personalized and challenging to standardize. The 2018 categorization system incorporated stage (severity) and grade (rate of progression and risk factors) to facilitate a multimodal diagnosis, transcending the previous chronic/aggressive dichotomy (144). This change is meant to improve patient care and make way for precision or personalized medicine. Prediction, prevention, personalization, and patient involvement are all components of new methods, such as the proposed P4 Periodontics model (143). Precision periodontics would use biomarkers and machine learning to divide patients into subgroups based on biological, bacterial, and lifestyle factors. Treatment choices would be made by Artificial Intelligence (AI) using detailed data, such as individual case reports, rather than relying solely on high-level evidence. However, the area is still very new, as no confirmed diagnostic biomarkers have been identified yet (145).

By disclosing disease activity, progression risk, and treatment response, miRNA signatures provide a biological foundation for customized periodontal therapy. Low miR-200a levels are associated with tissue degradation, whereas high levels of miR-146a, miR-155, and miR-223 are associated with active inflammation. Individuals who may benefit from regular treatment, need extra assistance, or are at high risk of rapid progression may be identified through patient stratification based on miRNA profiles (Grade C). Clinical confirmation is necessary, but miRNA signatures, in conjunction with AI, have the potential to shift periodontal care from a one-size-fits-all approach to genuinely individualized therapy (146).

Precision medicine tries to make treatments more effective by using a person’s unique genetic, biomarker, and behavioral traits. MiRNAs are becoming increasingly important because they help regulate disease and inflammation (147). Periodontitis is a complicated, noncommunicable disease that causes tooth loss. Salivary miRNAs show promise as noninvasive biomarkers for predicting disease progression, enabling early detection and tailored treatment (148).

Saliva is a crucial diagnostic medium for early illness diagnosis, risk assessment, and treatment monitoring due to its high quantities of proteins, enzymes, microbial components, and miRNAs (149). Point-of-care devices that assist precision dentistry and customized treatment are made possible by biosensors, lab-on-a-chip systems, and AI (150). For oral disorders, including cancer and periodontitis, genetic polymorphisms (such as IL-17) and salivary testing have been investigated; however, it remains difficult to validate diagnostic and predictive biomarkers (151).

Salivary miR-146a/b, miR-155, and miR-203 were measured in 24 diabetics and 29 healthy controls with and without periodontitis by Al-Rawi et al. (133). People with diabetes and/or periodontitis have increased expression of these miRNAs. These miRNAs may serve as non-invasive markers of periodontal health, as miR-155 was the strongest predictor of periodontitis in non-diabetic individuals, whereas miR-146a was the most predictive in diabetic patients (133).

Despite its potential, miRNA-based customized periodontal therapy has several drawbacks. Only small samples (24 patients with diabetes and 29 healthy controls) with cross-sectional designs were used in studies such as Al-Rawi et al. (133), making it impossible to conclude whether higher miRNA levels indicate current inflammation or predict the course of the illness. Due to the lack of standardized procedures for saliva collection, storage, and normalization, results across studies differ significantly, and environmental variables that affect miRNA expression—such as smoking, nutrition, stress, and socioeconomic status—are seldom controlled for. Stage and grade are included in the 2018 categorization scheme, but miRNA integration is still hypothetical and lacks established cutoffs for clinical judgment. Personalized miRNA-based periodontics will remain speculative unless extensive, long-term studies verify miRNA signatures and determine how to combine biological data with patient diversity.

Longitudinal studies are crucial, even though cross-sectional research has shown that miRNAs are linked to periodontitis in blood, GCF, and saliva. To transition periodontal treatment from reactive to proactive, targeted, and preventive therapy, salivary miRNA profiles must be translated into clinical practice (148, 151). Multidisciplinary initiatives and clinical research may verify salivary assays and revolutionize periodontal treatment despite obstacles, biomarker inconsistencies, standardization problems, and regulatory barriers (150).

## 7 Synergistic potential of combining stem cells with miRNA modulation

The integration of stem cells and miRNA modulation synergistically addresses critical challenges in stem cell therapy, including low cell viability, erratic differentiation, inadequate targeting, and variable paracrine signaling. Preconditioning stem cells with specific miRNA mimics or inhibitors enhances resistance to apoptosis, ensures adherence to defined developmental trajectories, and improves migratory efficacy toward wounded regions (152). This modulation simultaneously amplifies beneficial signals via EXOs that transport anti-inflammatory, angiogenic, and pro-regenerative miRNAs to adjacent tissues. Studies from 2023–2026 demonstrate that this integrated approach yields superior healing outcomes compared to stem cells or miRNAs alone. Current methodologies emphasize engineering stem cells to generate tailored, miRNA-enriched EVs as cell-free therapeutics that combine stem cell transport capabilities with the molecular specificity of miRNAs (153).

Human pulp tissue-derived stem cells (PSCs) are a good source of dental MSCs. They include hDPSCs from the dental pulp of permanent teeth and SHEDs. These cells could be used to rebuild the stomatognathic system and fix other damaged tissues (154). hDPSCs and SHEDs can repair damaged tissues by differentiating into osteogenic, odontogenic, myogenic, neurogenic, angiogenic, and immunomodulatory cells. The way miRNAs interact with their target genes can either help or stop the multi-lineage development of pluripotent stem cells. Modulating the expression of functional miRNAs in PSCs by blocking or mimicking them has become a useful therapeutic tool for clinical translation. A lot of focus has been placed on the safety and usefulness of miRNA-based therapeutics, as well as their greater stability, biocompatibility, and lower off-target effects and immunogenicity (155).

Combining stem cell therapy with miRNA modulation addresses two major challenges in periodontal treatment: impaired stem cell function in inflamed environments and the need for precise, long-term control of healing (156). EXOs generated from DFSCs preconditioned with L-D-EXO demonstrate enhanced therapeutic efficacy by specifically inhibiting miR-184 (157). Inhibiting this miRNA promotes the PPARα-Akt-JNK pathway, thereby diminishing oxidative stress, decreasing pro-inflammatory cytokines such as IL-6 and TNF-α, and enhancing the production of osteogenic markers such as Osteocalcin and RUNX2 in periodontal tissues. Advanced delivery mechanisms, such as tetrahedral framework nucleic acids encapsulating miR-200c (T-200c), have been developed to protect miRNAs from degradation and enhance their absorption by PDLSCs (158). This method slows NF-κB signaling while accelerating the Akt/β-catenin pathway. This restores PDLSC activity in inflammatory situations. Furthermore, miR-423-5p is an important miRNA in DFSC-EXOs. This has led to the creation of modified small EVs containing 100,000 times more miRNA. These bubble-like structures bind PLCB1, thereby initiating Wnt/β-catenin signaling. This promotes the growth of Sfrp2+ osteogenic fibroblasts, which in turn allows the PDL, bone, and cementum to heal almost completely (159).

Moreover, exosomal miR-21 originating from regulatory T cells has been shown to promote the osteogenic differentiation of PDLSCs and mitigate periodontal tissue damage *in vivo* (160). These findings collectively demonstrate that miRNA regulation transforms stem cell-derived vesicles from passive carriers into programmable, precise nanotherapeutics that can address the inflammatory problems of periodontitis and facilitate coordinated multi-tissue regeneration (160).

A study demonstrated that DSC-EXOs, made from DSCs, are a better, non-cellular alternative to root canal treatment for restoring living pulp tissue. Normal treatment weakens teeth and increases their susceptibility to infection. DSC-EXOs, on the other hand, deliver healing miRNAs and proteins that support odontoblast differentiation, blood vessel formation, nerve growth, and the control of inflammation. EXOs have been successfully used to restore tissue resembling pulp in animal studies. However, clinical translation is hindered by inconsistent isolation methods, donor variability, and poorly understood molecular pathways. So, the researchers have developed an integrated system that combines DSC-EXOs with scaffolds and dentine-derived signals to enable the functional, live regeneration of the pulp–dentine complex (161).

Combining stem cells with miRNA-based regulation for periodontal regeneration has several drawbacks, despite encouraging preclinical data. The majority of data comes from animal models lacking human validation, and inconsistent isolation techniques and donor variability hamper the reproducibility of EXO research. The optimal dosage, timing, and combination methods remain unknown; scalable, GMP-compliant manufacturing for clinical application has not been developed, and molecular pathways are still poorly understood. Standardized EXO isolation and characterization procedures, large-animal models of naturally occurring periodontitis, mechanistic clarification of miRNA-EXO interactions, GMP-compliant production, early-phase clinical trials, and optimization of combination strategies with scaffolds and growth factors should be the top priorities for future research.

## 8 Future direction

PD is a persistent inflammatory disorder affecting the supporting structures of the teeth, induced by an aberrant immune response to plaque-associated bacteria, notably *P. gingivalis* (162) (Kasurinen and Norvio). If left untreated, it advances from modest inflammation to significant tissue loss, encompassing alveolar bone damage and tooth dislocation. MiRNAs play a pivotal regulatory role in this process by modulating gene expression involved in inflammation, immune response, and tissue homeostasis (163). Expressed in periodontal progenitor cells (PDLSCs), GECs, and detectable in saliva and blood, miRNAs serve as potential biomarkers and therapeutic targets by influencing osteoclastogenesis and cytokine production (164).

To translate miRNA-based findings into therapeutic applications, several important goals need to be pursued. Before use in clinical settings, biomarker candidates such as miR-21-3p, miR-146a, miR-155, and miR-200 must be validated through extensive clinical trials (36). Meanwhile, EVs have potential as therapeutic targets and delivery systems, transporting specific miRNAs, mRNAs, and proteins between periodontal cells (165). Adipose-derived stem cell EXOs show better tissue regeneration than stem cells alone, whereas MSC-derived EXOs facilitate bone and PDL repair via AKT/ERK signaling (166). The biomarker value of consistently dysregulated miRNAs, such as miR-15a, miR-29b, miR-125a, miR-146a, miR-148/148a, and miR-223 (upregulated) and miR-92 (downregulated), is supported by their stability in GCF (167–170). With promising preclinical applications in immunological disorders, allergic airway illnesses, and anti-tumor immunotherapy (targeting miR-126, miR-145, miR-326, miR-138, and miR-155), miRNA mimics and antagomirs show therapeutic potential for controlling inflammation and targeting numerous genes (169, 170).

Despite encouraging preclinical results, clinical translation is hampered by several crucial gaps. The majority of interesting miRNA candidates have not been validated in extensive clinical studies, and it is necessary to determine if GCF includes miRNAs and their biomarker value (71). Cell-type-specific miRNA activities and EV-mediated intercellular communication require further mechanistic clarification, and the optimal medication combinations, doses, and delivery methods remain unknown. To make miRNA-based diagnoses and treatments clinically feasible, it will be crucial to close these gaps through extensive longitudinal cohort studies, standardized procedures, and thorough functional validation.

An integrated research agenda is proposed to expedite the clinical translation of miRNA-based periodontitis treatment, informed by the thorough study. While therapeutic optimization should concentrate on miRNA mimics (miR-146a, miR-27, miR-210) and antagomirs (miR-338-3p, miR-155, miR-223) using cutting-edge delivery systems (LNPs, hydrogels, EXOs) in large-animal models, followed by early-phase trials, diagnostic biomarker development necessitates large-scale longitudinal validation of salivary and GCF miRNA panels with established clinical cutoffs. In addition to AI-driven patient stratification algorithms that integrate miRNA signatures with clinical, genetic, and environmental variables, combination techniques combining miRNA treatment with stem cells, scaffolds, and growth factors should be investigated. Establishing the molecular foundation and clinical feasibility of miRNA-based periodontal therapies requires long-term safety studies (6–12 months), GMP-compliant production, standardized techniques, single-cell miRNA analysis, and clarification of EV-mediated intercellular communication.

To stop PD from progressing, future research will focus on identifying the optimal treatment combinations, doses, and delivery systems (169, 170) (**Table 6**).

**Table 6:** Summary of challenges and future directions for miRNA-based periodontitis therapeutics.

Category	Specific challenge	Proposed solution/future direction	REF
Stability	Rapid degradation by nucleases; short half-life	Chemical modifications (locked nucleic acids); encapsulation in hydrogels, LNPs, or EXOs	(114, 119–121)
Cellular uptake	Poor intracellular delivery; endosomal entrapment	Cell-penetrating peptides; ligand-targeted NPs; EXO-based carriers	(114, 133)
Off-target effects	Single miRNA regulates dozens to hundreds of genes	Genome-wide off-target analysis; precise delivery systems; stringent seed region matching	(120, 121, 133)
Immunotoxicity	TLR activation; neurotoxicity	Biocompatible carriers (hydrogels, EXOs); chemically modified RNAs	(114, 118)
Patient variability	Diverse miRNA profiles due to smoking, diabetes, and genetics	Personalized miRNA profiling; AI-driven patient stratification; P4 Periodontics model	(126, 132, 133)
Model limitations	Small animal models (mice, rats) don’t mimic human CP	Large animal models (minipigs, dogs) with naturally occurring periodontitis	(117, 124)
Short follow-up	Studies limited to 4–8 weeks	Long-term safety studies (6–12 months)	(117, 123)
Standardization	No standard methods for miRNA isolation, normalization, or dosing	Establish standardized protocols; GMP-compliant manufacturing	(8, 127)
Comparison to gold standards	Lack of comparison with Emdogain, GTR membranes, scaling/root planing	Direct head-to-head preclinical and clinical comparisons	(114, 133)
Combination therapy	Single miRNA targeting may be insufficient.	Combination therapies targeting multiple pathogenic miRNAs (*e.g.*, miR-210 mimic + miR-16 inhibitor)	(117, 133)
Biodistribution	Unclear systemic distribution and potential toxicity	Comprehensive biodistribution studies; local delivery systems	(122, 123)
EXO challenges	Inconsistent isolation; low native miRNA cargo	Engineered EXOs with enhanced miRNA loading; standardized production	(124)

## 9 Conclusion

PD is not solely attributable to bacteria; it is fundamentally influenced by the immune system’s response to these bacteria, which ultimately leads to the degradation of the bone supporting these teeth. This review clearly indicates that miRNAs are crucial to that process. These small molecules regulate the behavior of human immune cells (such as neutrophils, macrophages, and T cells) by promoting or mitigating inflammation, and they determine whether stem cells in the PDL can differentiate into osteogenic cells. Certain miRNAs, notably miR-146a, miR-155, miR-223, miR-21, and the let-7 family, are continuously dysregulated in periodontitis and may be detected in saliva and gingival fluid. This implies they may eventually serve as straightforward, non-invasive assessments for detecting the disease early or monitoring therapy efficacy. Preliminary investigations indicate significant potential in treatment: inhibiting detrimental miRNAs with antagomirs or enhancing beneficial ones with mimics can mitigate bone loss, alleviate inflammation, and facilitate tissue regeneration. Innovative delivery technologies such as hydrogels, LNPs, and EXOs are enhancing the stability and specificity of these medicines. However, researchers must acknowledge that we have not yet arrived at that point. Significant challenges persist: miRNAs degrade rapidly throughout the body, may inadvertently inhibit the wrong genes, and each patient’s miRNA profile varies based on factors such as smoking, diabetes, and certain genetic characteristics. We also lack standardized methods for measuring miRNAs, and most studies have been conducted only in mice, rather than in larger animals or people. No miRNA therapeutic has successfully progressed beyond Phase II clinical trials for any illness. In the future, we require further investigation, including larger animal models, more sophisticated delivery methods that target specific cells, and individualized strategies that use AI to align each patient with the appropriate miRNA-based therapy. The essential conclusion is that miRNA-based diagnostics and therapeutics have significant potential to transform PD treatment; nonetheless, their transition from the laboratory to dental practice will require interdisciplinary collaboration and considerable effort.
